# Optimization of 4-Anilinoquinolines as Dengue Virus Inhibitors

**DOI:** 10.3390/molecules26237338

**Published:** 2021-12-03

**Authors:** Pei-Tzu Huang, Sirle Saul, Shirit Einav, Christopher R. M. Asquith

**Affiliations:** 1 Department of Medicine, Division of Infectious Diseases and Geographic Medicine, Stanford University School of Medicine, Stanford, CA 94305, USA; pthuang@stanford.edu (P.-T.H.); sauls@stanford.edu (S.S.); 2 Department of Microbiology and Immunology, Stanford University School of Medicine, Stanford, CA 94305, USA; 3 Department of Pharmacology, School of Medicine, University of North Carolina at Chapel Hill, Chapel Hill, NC 27599, USA; 4 Structural Genomics Consortium, UNC Eshelman School of Pharmacy, University of North Carolina at Chapel Hill, Chapel Hill, NC 27599, USA

**Keywords:** dengue virus (DENV), 4-anilinoquinoline, flavivirus, alphavirus, VEEV, antivirals, emerging viruses

## Abstract

Emerging viral infections, including those caused by dengue virus (DENV) and Venezuelan Equine Encephalitis virus (VEEV), pose a significant global health challenge. Here, we report the preparation and screening of a series of 4-anilinoquinoline libraries targeting DENV and VEEV. This effort generated a series of lead compounds, each occupying a distinct chemical space, including 3-((6-bromoquinolin-4-yl)amino)phenol (**12**), 6-bromo-*N*-(5-fluoro-1H-indazol-6-yl)quinolin-4-amine (**50**) and 6-((6-bromoquinolin-4-yl)amino)isoindolin-1-one (**52**), with EC_50_ values of 0.63–0.69 µM for DENV infection. These compound libraries demonstrated very limited toxicity with CC_50_ values greater than 10 µM in almost all cases. Additionally, the lead compounds were screened for activity against VEEV and demonstrated activity in the low single-digit micromolar range, with **50** and **52** demonstrating EC_50_s of 2.3 µM and 3.6 µM, respectively. The promising results presented here highlight the potential to further refine this series in order to develop a clinical compound against DENV, VEEV, and potentially other emerging viral threats.

## 1. Introduction

Mosquito-borne viral infections, including those caused by the flavivirus dengue (DENV) and the alphavirus Venezuelan Equine Encephalitis virus (VEEV), represent a major public health concern [1,2,3,4]. Rapid urbanization and climate change have contributed to the expanding geographical range of DENV infections into the developed world [5] and have caused a significant increase in the number of annual infections, currently estimated at ~400 million people in over 128 endemic countries [6,7]. The majority of symptomatic DENV infected patients experience mild illness, yet approximately 5–20%, particularly those with a secondary infection with a heterologous DENV serotype, progress into a potentially life-threatening disease known as severe dengue [8,9]. Currently, there are no approved antiviral therapies for DENV infection, and the development of an effective and safe DENV vaccine has been challenged by the need to generate a balanced protective immunity against the four distinct DENV serotypes.

The alphavirus VEEV is an important causative agent of neurological disease in Central and South America [10]. While most VEEV infections in humans are mild, approximately 14% of the patients develop encephalitis, often complicated by severe neurological deficits and sometimes death [11]. Beyond mosquito bites, VEEV can be transmitted via aerosol exposure and is considered a major bioterrorism threat, underscoring the importance of developing effective countermeasures [12]. However, there are currently no approved antiviral drugs nor licensed human vaccines available for VEEV infection. In the absence of approved antiviral therapies for DENV and VEEV infections, the management of infected patients largely relies on supportive care [12,13], resulting in continued morbidity and mortality. 

**Figure 1:**
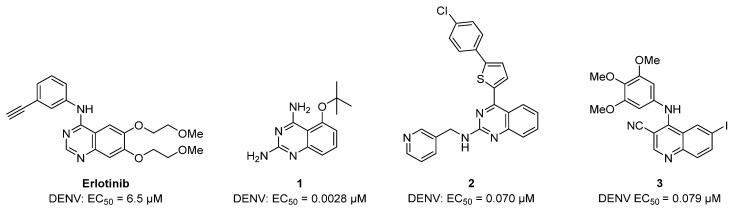
A selection of previously reported quinazolines and quinolines with anti-DENV activity (the data is all for serotype 2 for consistency).

In the past decade, there has been a significant effort to develop small molecules for the treatment of DENV infection, yielding multiple promising candidates with a number of different scaffolds. [14,15,16,17,18,19,20,21,22,23,24,25,26,27,28,29,30,31,32,33] However, none of these compounds have entered clinical trials for DENV treatment. Interestingly, many of these compounds include common kinase inhibitor scaffolds, such as the oxindole [14], *iso*thiazolo[4,3-*b*]pyridine [15] and azaindoles [16]. Another kinase scaffold with reported anti-DENV activity has been the 4-anilinoquin(az)oline, present in a number of clinically approved kinase inhibitors [34,35,36,37]. This includes erlotinib, which has shown low micromolar activity against DENV, and several other, lower molecular weight analogues of the same scaffold (**1**–**3**), showing effective, low nanomolar activity against DENV (Figure 1) [14,29,30,31,32,33]. 

## 2. Results

### 2.1. Synthesis

**Figure 2:**
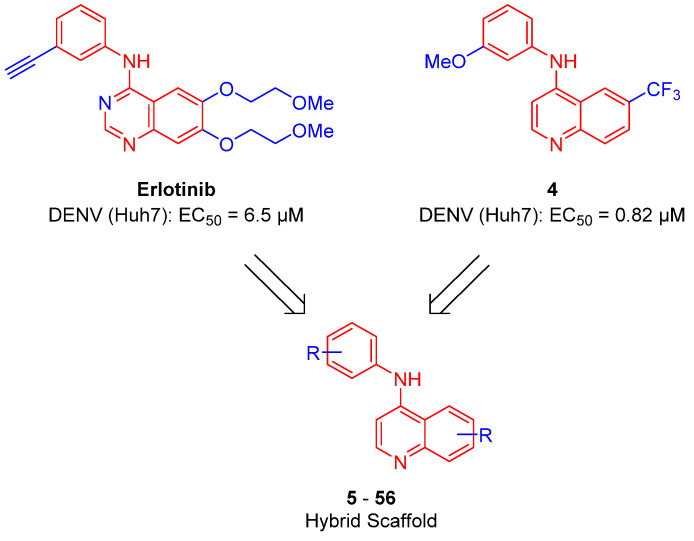
Design of a hybrid series of 4-anilinoquinolines developed by combining erlotinib and **4**.

Building on our previous work, to further explore the antiviral activity of the 4-anilinoquin(az)oline [14,32,33], we probed the structure activity relationships (SAR) with a series of focused libraries. We developed a series of hybrid molecules combining structural features of erlotinib and lead compound **4** to enhance the SAR in the current literature and improve the tractability of the quin(az)oline scaffold (Figure 2).

**Scheme 1:**
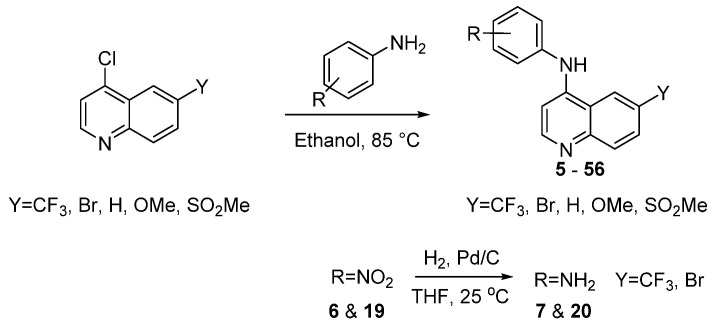
General synthetic procedure to access the 4-anilinoquinolines.

We hence synthesized a series of compounds (**5**–**56**) to create the hybrid structure of erlotinib and **4**. We accessed these 4-anilinoquinolines through nucleophilic aromatic displacement of 4-chloroquin(az)olines with the respective aniline (Scheme 1) [38,39,40,41,42,43,44,45,46,47,48]. We were able to access products in full range of yields, from moderate to excellent (21–94%), consistent with previous reports for literature compounds [38,39,40,41,42,43,44,45,46,47,48].

### 2.2. Antiviral Screening

To evaluate the compounds for broad-spectrum antiviral activity we first studied their effect on DENV2-Rluc virus infection, a wild type virus carrying a *Renilla* luciferase reporter gene (virus production described in Materials and Methods). We measured the effect of compound treatment on overall infection in human hepatoma (Huh7) cells 48 h following infection with DENV2 via luciferase assays and calculated the half-maximal effective concentration (EC_50_) relative to DMSO treated cells. In parallel, we tested the effect of these compounds on cell viability via an AlamarBlue assay in the DENV-infected Huh7 cells and measured the half-maximal cytotoxic concentration (CC_50_) values relative to DMSO treated cells.

In order to follow-up on the lead compound **4**, we first screened a series of focused substitutions at the aniline *meta*-position (**5**–**10**) (Table 1) [48]. The hydroxy analogue **5** was 3-fold less potent than the methoxy **4**. The nitro substitution **6** was inactive at the top concentration, while the reduced amine **7**, was 7-fold weaker with respect to **4** and 2-fold weaker with respect to the direct isosteric replacement **5**. The monomethyl substituted amine **8** showed no improvement, while the dimethyl substitution showed a 2-fold increase in potency against DENV with respect to **7**. The methanolic substitution **10**, was 2-fold weaker with respect to **5** and 8-fold with respect **4**.

We then screened a series of matched pair *meta*-methoxy and *meta*-hydroxy analogues (**11**–**18**), changing the 6-position of the quinoline ring (Table 2) [48]. The 6-bromoquinoline methoxy analogue **11** was equipotent with the 6-triflurormethyl quinoline methoxy analogue **4**. However, switching to the hydroxy **12** led to a 4-fold increase in potency against DENV (EC_50_ = 0.63 μM). The unsubstituted quinoline methoxy analogue **13** showed a 3-fold decrease in activity against DENV, while the hydroxy analogue **14** showed no activity at the top concentration tested. Switching to the electron donating 6-methoxyquinoline methoxy analogue **15** was 5-fold less potent with respect to **4**, while the hydroxy analogue was inactive (EC_50_ = >10 μM). Interestingly, both the 6-methylsulfone quinoline analogues **17** and **18** were inactive.

Encouraged by the result of 3-((6-bromoquinolin-4-yl)amino)phenol **12,** we followed up with a series of focused analogues (**19**–**31**) fixing the 6-bromoquinoline and altering the aniline ring substituent (Table 3) [48]. The direct nitro analogue **19** and reduced amine version **20** were both inactive (EC_50_ = >10 μM). The substitution of the amine **20** with a methyl group to afford **21** produced a compound with a potent antiviral activity profile (EC_50_ = 0.24 μM), but this appeared to be in part driven by toxicity (CC_50_ = 5.3 μM) with a 22-fold selectivity index window. This toxicity interference appeared more likely considering **19** and **20** were inactive, as was the dimethyl analogue **22**. The methanol analogue **23** was 6-fold weaker with respect to **12** and the *ortho*-position methanol analogue **24** a further 2-fold weaker with respect to **23**. We then tested the scope of the *meta*-position further with several bulkier substituents (**25**–**27**). The pentafluorosulfanyl **25** was just over 2-fold less potent than **12**, demonstrating that there is potentially room for expansion at this position [49]. The *tert*-butoxy **26** 2-fold less potent than **12**, with the *tert*-butyl **27** near equipotent (EC_50_ = 0.94 μM), unfortunately both showed a level of toxicity (CC_50_ = 9.2 μM and CC_50_ = 6.0 μM respectively). We then explored how torsional strain on the methoxy orientation would affect activity (**28**–**31**). The fused ring systems were overall less active against DENV, only **28** showed activity (EC_50_ = 1.7 μM) which was accompanied by light toxicity (CC_50_ = 8.6 μM).

Predicting that we would identify active analogues based on the 2,3- and 3,4-connectivty, we followed up on the results of **28**–**31** with a series of different substitutions and fused heterocycles (**32**–**56**) (Table 4) [42]. The introduction of a fluorine to the distal ortho-position on the aniline ring of **30** produced compound **32** with good activity against DENV (IC_50_ = 1.9 μM). The ring opened version of **30**, **33** was 4-fold weaker against DENV. The substitution of the catechol carbon linker of **30** with a gem-difluoro **34** afforded a compound with equipotency with the respective ring opened analogue **33** but was slightly more active than the parent **30**. The removal of the *ortho*-substituted oxygen of **30** furnished **35**, a compound with sub-micromolar activity against DENV (EC_50_ = 0.95 μM). However, the removal of the *meta*-substituted oxygen of **30** to afford **36** led to a 6-fold decrease in potency compared with **35**. Aromatising **35** to yield **37** led to a 3-fold decrease in potency (EC_50_ = 2.7 μM), whereas aromatising **36** to afford **38** demonstrated a 3-fold increase in potency (EC_50_ = 1.9 μM). The formation of the respective isoxazole’s of **37** and **38**, affording **39** and **40,** produced inactive compounds at the top concentration tested (EC_50_ = >10 μM). Moving the nitrogen of **39** to form the oxazole **41** led to a >3-fold increase in potency (EC_50_ = 3.2 μM). The reversed thiazole **42**, was not active (EC_50_ = >10 μM), but removal of the nitrogen from the thiazole ring system afforded a thiophene **43** that had equipotent activity with oxazole **41**. 

A furazan substitution **44** only showed limited activity against DENV (EC_50_ = 9.2 μM), whereas the introduction of a sulphur to construct a 1,2,5-thiadiazole **45** led to a 2-fold increase in potency compared with **44**. The introduction of a methyl distal to the quinoline ring system via a 1-methyltriazole **46** or 1-methyl-1*H*-pyrazole **47** led to compounds with approximately a 5 micromolar EC_50_ efficacy against DENV. The removal of the methyl of **47** led to a >2-fold increase against DENV; interestingly, the reversed pyrazole (2,3 vs. 1,2) **49** was equipotent with **48**. The introduction of a fluorine in the distal *ortho*-position of the aniline in **49** produced compound **50** and led to an almost 3-fold increase against DENV with efficacy exceeding 1 micromolar (EC_50_ = 0.69 μM). Moving the pyrazole from 2,3- to the 3-4-postion; **48** to **51** led to a 2-fold boost in efficacy (EC_50_ = 1.2 μM). An increase in diversity to produce 6-((6-bromoquinolin-4-yl)amino)isoindolin-1-one (**52**) led to an equipotent inhibitor compared with **49,** though occupying a different chemical space. Several attempts to increase potency with 1,3-dihydro-2*H*-benzo[*d*]imidazol-2-one derivatives (**53**–**55**) and a fused cyclic sulfone (**56**) yielded compounds with efficacy only in the high micromolar range.

### 2.3. Extension Screening of Lead Compounds 

The parental compound (**4**) and the lead compounds (**12**, **21**, **50**, **52**) were then screened against VEEV (Table 5) [17]. We infected U-87 MG (human astrocytes) cells with the VEEV vaccine strain (TC-83) carrying a nanoluciferase reporter gene (virus production described in Materials and Methods) and measured the effect of compounds on viral replication via luciferase assays and cell viability via AlamarBlue assay at 18 h post-infection. The parental compound **4** was found to be inactive against VEEV at the top concentration tested. The 3-((6-bromoquinolin-4-yl)amino)phenol analogue **12** was also weakly active (EC_50_ = 9.9 μM). However, the *N*^1^-(6-bromoquinolin-4-yl)-*N*^3^-methylbenzene-1,3-diamine **21** showed good activity (EC_50_ = 1.0 μM) without the associated mild toxicity observed in Huh7 cells. Compounds **50** and **52** also both performed well with a low micromolar potency against VEEV (EC_50_ = 2.3 μM and 3.6 μM, respectively). Consistent with our previous reports, the antiviral effect of sunitinib was in the submicromolar range [17,36]. As an additional toxicity control, we screened these lead compounds for their effect on cellular viability in Vero (African green monkey kidney epithelial) cells [50] and found no toxicity at the top concentration tested (10 μM). 

**Figure 3:**
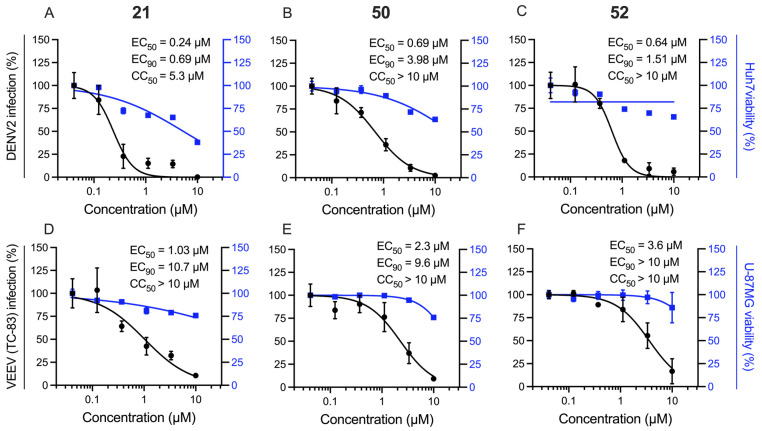
Compounds **21**, **50** and **52** suppress both DENV2 and VEEV (TC-83) infection. Dose response of DENV2 (black) and cell viability (blue) to compounds A: **21**, B: **50**, C: **52** and dose response of VEEV (TC-83) (black) and cell viability (blue) to compounds D: **21**, E: **50**, F: **52**; each measured by luciferase and AlamarBlue assays, 48 h post-infection (**A**–**C**) or 18 h post-infection (**D**–**F**). Data are plotted relative to DMSO control. Shown are representative experiments from at least two conducted, each with 4 biological replicates; shown are means ± SD.

The lead compounds against DENV include 3-((6-bromoquinolin-4-yl)amino)phenol (**12**) (EC_50_ = 0.63 µM), 6-bromo-*N*-(5-fluoro-1H-indazol-6-yl)quinolin-4-amine (**50**) (EC_50_ = 0.69 µM) and 6-((6-bromoquinolin-4-yl)amino)isoindolin-1-one (**52**) (EC_50_ = 0.64 µM) (Figure 3). Each of these compounds occupy a distinct chemical space and enhances our understanding of the SAR within this series. We also identified *N*^1^-(6-bromoquinolin-4-yl)-*N*^3^-methylbenzene-1,3-diamine (**21**) with potent activity (EC_50_ = 0.24 µM), but some associated toxicity (CC_50_ = 5.3 µM) in the same Huh7 cell line (Figure 3). This toxicity was not observed in the Vero cell line secondary screening (CC_50_ = >10 µM). Compounds **21**, **50** and **52** all performed well against VEEV albeit with a 4-8-fold reduction in potency relative to DENV (Figure 3). Interestingly, **4** and **12** showed weak activity toward VEEV despite a good anti-DENV activity. 

## 3. Discussion

We have previously demonstrated that the 4-anilinoquinoline/quinazoline scaffold is active on both DENV and VEEV [32,33]. The current results re-enforce our interest in the 4-anilinoquin(az)oline scaffold, with the relatively low molecular weight compound **12** showing good potency (EC_50_ = 0.63 µM). Despite some mild toxicity (EC_50_ = 5.3 µM), the direct methylamine analogue **21** was even more potent (EC_50_ = 0.24 µM) albeit with a reduced therapeutic index. The other two lead compounds identified were 6-bromo-*N*-(5-fluoro-1H-indazol-6-yl)quinolin-4-amine (**50**) (EC_50_ = 0.69 µM) and 6-((6-bromoquinolin-4-yl)amino)isoindolin-1-one (**52**) (EC_50_ = 0.64 µM), both of which are isosteric replacements for the original trimethoxy of **3** and related to 3-methoxy of **4**. Three of our four lead compounds (**21**, **50** and **52**) have shown promising antiviral activity against both DENV and VEEV and offer interesting insights into the SAR within this series. 

While the mechanism of the antiviral activity on both viruses is being investigated, potentially it could be targeting multiple protein targets including both cellular kinases and other proteins. Several of these compounds were originally prepared as inhibitors targeting the ATP-binding site of human kinases including cyclin-G-associated kinase (GAK), serine/threonine-protein kinase 10 (STK10) and STE20-like serine/threonine-protein kinase (SLK) [42,47,48]. While our compounds could be targeting these kinases, it is also possible that other undefined proteins may participate in the mechanism of antiviral action. Multiple examples from previous literature show that compounds with structural features impeding interaction with a kinase hinge region, have antiviral activity [35]. It is also possible that the observed phenotypes may originate from modulation of other kinases or non-ATP binding proteins [51]. Lastly, beyond targeting cellular kinases, it is possible that these compounds target a viral protein. Indeed, related quinolinones were identified as VEEV inhibitors targeting the viral nonstructural protein (nsP2) [52,53]. 

This set of results presents a potential step towards the identification of a novel clinical compound to combat emerging viral diseases including DENV and VEEV infections and provides a medicinal chemistry trajectory to achieve this aim. In addition, several quinoline derivatives have been shown to inhibit alpha- and beta-coronaviruses [54], demonstrating the promising potential of quinoline group as part of a broader spectrum antiviral compound. 

## 4. Materials and Methods

### 4.1. Chemistry Method

#### General Procedure for the Synthesis of 4-Anilinoquin(az)olines

We suspended 4-chlo-quin(az)oline derivative (1.0 eq.), aniline derivative (1.1 eq.), in ethanol (10 mL) and refluxed for 18 h. The crude mixture was purified by flash chromatography using EtOAc:hexane followed by 1–5% methanol in EtOAc; After solvent removal under reduced pressure, the product was obtained as a free following solid or recrystallized from ethanol/water. Compounds **4**–**31** were synthesized as previous described [38,48], 32-51 were synthesized as previously described [42]. Representative supporting information provided (see Supplementary Materials).

### 4.2. Antiviral Screening

#### 4.2.1. Virus Construct

DENV2 (New Guinea C strain) [55,56] *Renilla* reporter plasmid used for virus production (DENV2-Rluc) was a gift from Pei-Yong Shi (The University of Texas Medical Branch). The plasmid encoding infectious VEEV (TC-83) with a nanoluciferase reporter and used for virus production (VEEV-TC-83-nLuc) was a gift from Dr. William B. Klimstra (Department of Immunology, University of Pittsburgh) [57].

#### 4.2.2. Cells

Huh7 (Apath, L.L.C, New York, NY, USA) cells were grown in Dulbecco’s Modified Eagle’s medium (DMEM) (10-013-CV: Corning, New York, NY, USA) supplemented with 10% fetal bovine serum (FBS) (Omega Scientific, Tarzana, CA, USA), nonessential amino acids, 1% L-glutamine, and 1% penicillin-streptomycin (Thermo Fisher Scientific, Waltham, MA, USA). U-87 MG cells obtained from ATCC were grown in DMEM supplemented with 10% FBS and 1% penicillin-streptomycin solution. Cells were maintained in a humidified incubator with 5% CO_2_ at 37 °C. 

#### 4.2.3. Virus Production

DENV2-Rluc RNA was transcribed in vitro using mMessage/mMachine (Ambion Austin, TX, USA) kits. DENV2-Rluc virus was produced by electroporating RNA into BHK-21 cells, harvesting supernatants on day 10 post-electroporation and titering via standard plaque assays on BHK-21 cells. VEEV-TC-83-nLuc RNA was transcribed in vitro from cDNA plasmid template linearized with MluI via MegaScript Sp6 kit (Invitrogen #AM1330, Thermo Fisher Scientific, Waltham, MA, USA) and electroporated into BHK-21 cells. The VEEV-TC-83-nLuc virus was harvested from the supernatant 24 h post-electroporation, clarified from cell debris and the titer determined by standard plaque assay on Vero cells.

#### 4.2.4. Infection Assays

Huh7 cells were treated with the inhibitors or DMSO. An hour later, the cells were infected with DENV2-Rluc virus in replicates (*n* = 5) at a multiplicity of infection (MOI) of 0.05. The inhibitors were present for the duration of the experiment. Overall infection was measured at 48 h post-infection using a Renilla luciferase substrate. U-87 MG cells were treated with the inhibitors or DMSO. One hour later, the cells were infected with VEEV-TC-83-nLuc virus in 5 replicates at MOI of 0.01, and overall infection was measured at 18 h post-infection via a nanoluciferase assay. The inhibitors were present for the duration of the experiment. The relative light units (RLUs) were normalized to DMSO treated cells (set as 100%). GraphPad Prism nonlinear regression (curve fit) was used to generate the graphs and EC_50_ values. 

#### 4.2.5. Viability Assays

Viability was measured using AlamarBlue^®^ reagent (Thermo Fisher Scientific, Waltham, MA, USA) according to the manufacturer’s protocol. Fluorescence was detected at 560 nm on InfiniteM1000 plate reader (Tecan, Männedorf, Switzerland). The raw fluorescence values were normalized to DMSO treated cells (set as 100%). Graphpad Prism nonlinear regression (curve fit) was used to generate the graphs and CC_50_ values. 

## Figures and Tables

**Table 1:**
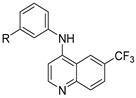
Screening results of anti-DENV activity of initial SAR around 3-methoxy aniline substitution pattern of **4**.

Cmpd	R	DENV Inhibition	Cell Viability
		EC_50_^a^	CC_50_^b^
**4**	OMe	0.82	>10
**5**	OH	2.7	>10
**6**	NO_2_	>10	>10
**7**	NH_2_	5.9	>10
**8**	NHMe	8.9	>10
**9**	NMe_2_	2.3	>10
**10**	CH_2_OH	6.6	>10

^a^ = infectivity assay in Huh7 cells mean average *n* = 5; ^b^ = cytotoxicity in Huh7 cells mean average *n* = 5.

**Table 2:**
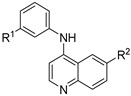
Screening results of anti-DENV activity of matched pair methoxy and hydroxy aniline analogues.

Cmpd	R^1^	R^2^	DENV Inhibition	Cell Viability
			EC_50_^a^	CC_50_^b^
**4**	OMe	CF_3_	0.82	>10
**5**	OH	CF_3_	2.7	>10
**11**	OMe	Br	0.84	>10
**12**	OH	Br	0.63	>10
**13**	OMe	H	2.5	>10
**14**	OH	H	>10	>10
**15**	OMe	OMe	4.4	>10
**16**	OH	OMe	>10	>10
**17**	OMe	SO_2_Me	>10	>10
**18**	OH	SO_2_Me	>10	>10

^a^ = infectivity assay in Huh7 cells mean average *n* = 5; ^b^ = cytotoxicity in Huh7 cells mean average *n* = 5.

**Table 3:**
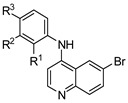
Screening results of anti-DENV activity of analogues of **12**.

Cmpd	R^1^	R^2^	R^3^	DENV Inhibition	Cell Viability
				EC_50_^a^	CC_50_^b^
**12**	H	OH	H	0.63	>10
**19**	H	NO_2_	H	>10	>10
**20**	H	NH_2_	H	>10	>10
**21**	H	NHMe	H	0.24	5.3
**22**	H	NMe_2_	H	>10	>10
**23**	H	CH_2_OH	H	3.7	>10
**24**	CH_2_OH	H	H	7.1	>10
**25**	H	SF_5_	H	1.5	>10
**26**	H	O^t^Bu	H	2.3	9.2
**27**	H	^t^Bu	H	0.94	6.0
**28**	H	OCH_2_O		1.7	8.6
**29**	H	CH_2_OCH_2_		>10	>10
**30**	OCH_2_O		H	>10	>10
**31**	CH_2_OCH_2_		H	>10	>10

^a^ = infectivity assay in Huh7 cells mean average *n* = 5; ^b^ = cytotoxicity in Huh7 cells mean average *n* = 5.

**Table 4:** Screening results of anti-DENV activity following up on **28**–**31**.

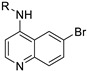				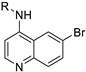			
Cmpd	R	DENV Inhibition	Cell Viability	Cmpd	R	DENV Inhibition	Cell Viability
		EC_50_^a^	CC_50_^b^			EC_50_^a^	CC_50_^b^
**32**	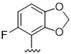	1.9	>10	**45**	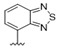	5.8	>10
**33**	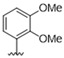	7.8	>10	**46**	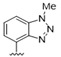	5.5	>10
**34**	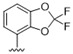	7.1	>10	**47**	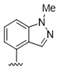	5.4	>10
**35**	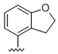	0.95	>10	**48**	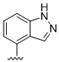	2.1	>10
**36**	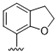	5.7	>10	**49**	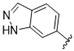	1.8	>10
**37**	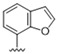	2.7	>10	**50**	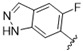	0.69	>10
**38**	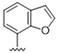	1.9	>10	**51**	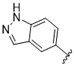	1.2	>10
**39**	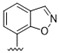	>10	>10	**52**	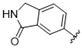	0.64	>10
**40**	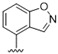	>10	>10	**53**	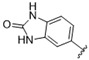	>10	>10
**41**	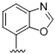	3.2	>10	**54**	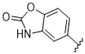	>10	>10
**42**	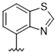	>10	>10	**55**	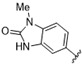	7.1	>10
**43**	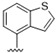	2.9	>10	**56**	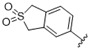	6.3	>10
**44**	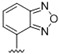	9.2	>10	-	-	-	-

^a^ = infectivity assay in Huh7 cells mean average *n* = 5; ^b^ = cytotoxicity in Huh7 cells mean average *n* = 5.

**Table 5:** Extension screening on VEEV and on Vero cells for lead compounds **4**, **12**, **21**, **50** and **52**.

Cmpd	DENV Inhibition ^a^			VEEV Inhibition ^b^			Vero Cells ^c^
	EC_50_	EC_90_	CC_50_	EC_50_	EC_90_	CC_50_	CC_50_
**4**	0.82	1.5	>10	>10	>10	>10	>10
**12**	0.63	3.2	>10	9.9	>10	>10	>10
**21**	0.24	0.69	5.3	1.0	>10	>10	>10
**50**	0.69	4.0	>10	2.3	9.6	>10	>10
**52**	0.64	1.5	>10	3.6	>10	>10	>10
**Sunitinib**	1.1	>10	>10	4.4	>10	>10	>10

^a^ = infectivity assay in Huh7 cells mean average *n* = 5; cytotoxicity in Huh7 cells mean average *n* = 5; ^b^ = infectivity assay in U-87 MG cells mean average *n* = 5; cytotoxicity in U-87 MG cells mean average *n* = 5; ^c^ = cytotoxicity 48 h in Vero cells.

## Data Availability

The data presented in this study are available in Supplementary Material.

## Supplementary Materials

The following are available online, representative LC-HRMS, ^1^H and ^13^C NMR spectra on the lead compound compounds.

## Sample Availability

Samples of the compounds are available from the authors.

## References

[1] Paixão E.S., Teixeira M.G., Rodrigues L.C. (2017). Zika, chikungunya and dengue: The causes and threats of new and re-emerging arboviral diseases. BMJ Glob. Health.

[2] Pierson T.C., Diamond M.S. (2020). The continued threat of emerging flaviviruses. Nat. Microbiol..

[3] Braack L., de Almeida A.P.G., Cornel A.J., Swanepoel R., de Jager C. (2018). Mosquito-borne arboviruses of African origin: Review of key viruses and vectors. Parasit Vectors.

[4] Silva N.M., Santos N.C., Martins I.C. (2020). Dengue and Zika Viruses: Epidemiological History, Potential Therapies, and Promising Vaccines. Trop. Med. Infect. Dis..

[5] Campbell L.P., Luther C., Moo-Llanes D., Ramsey J.M., Danis-Lozano R., Peterson A.T. (2015). Climate change influences on global distributions of dengue and chikungunya virus vectors. Philos. Trans. R. Soc. Lond. B Biol. Sci..

[6] Murray N.E., Quam M.B., Wilder-Smith A. (2013). Epidemiology of dengue: Past, present and future prospects. Clin. Epidemiol..

[7] Guzman M.G., Gubler D.J., Izquierdo A., Martinez E., Halstead S.B. (2016). Dengue infection. Nat. Rev. Dis. Primers.

[8] Flipse J., Smit J.M. (2015). The Complexity of a Dengue Vaccine: A Review of the Human Antibody Response. PLoS Negl. Trop. Dis..

[9] Yang Y., Meng Y., Halloran M.E., Longini I.M. (2018). Dependency of Vaccine Efficacy on Preexposure and Age: A Closer Look at a Tetravalent Dengue Vaccine. Clin. Infect. Dis..

[10] Sharma A., Knollmann-Ritschel B. (2019). Current Understanding of the Molecular Basis of Venezuelan Equine Encephalitis Virus Pathogenesis and Vaccine Development. Viruses.

[11] Guzmán-Terán C., Calderón-Rangel A., Rodriguez-Morales A., Mattar S. (2020). Venezuelan equine encephalitis virus: The problem is not over for tropical America. Ann. Clin. Microbiol Antimicrob..

[12] Hawley R.J., Eitzen E.M. (2001). Biological weapons—A primer for microbiologists. Annu. Rev. Microbiol..

[13] Stevens A.J., Gahan M.E., Mahalingam S., Keller P.A. (2009). The medicinal chemistry of dengue fever. J. Med. Chem..

[14] Bekerman E., Neveu G., Shulla A., Brannan J., Pu S.Y., Wang S., Xiao F., Barouch-Bentov R., Bakken R.R., Mateo R. (2017). Anticancer kinase inhibitors impair intracellular viral trafficking and exert broad-spectrum antiviral effects. J. Clin. Investig..

[15] Pu S.Y., Wouters R., Schor S., Rozenski J., Barouch-Bentov R., Prugar L.I., O’Brien C.M., Brannan J.M., Dye J.M., Herdewijn P. (2018). Optimization of Isothiazolo[4,3-b]pyridine-Based Inhibitors of Cyclin G Associated Kinase (GAK) with Broad-Spectrum Antiviral Activity. J. Med. Chem..

[16] Verdonck S., Pu S.Y., Sorrell F.J., Elkins J.M., Froeyen M., Gao L.J., Prugar L.I., Dorosky D.E., Brannan J.M., Barouch-Bentov R. (2019). Synthesis and Structure-Activity Relationships of 3,5-Disubstituted-pyrrolo[2,3-b]pyridines as Inhibitors of Adaptor-Associated Kinase 1 with Antiviral Activity. J. Med. Chem..

[17] Nitsche C., Steuer C., Klein C.D. (2011). Arylcyanoacrylamides as inhibitors of the Dengue and West Nile virus proteases. Bioorg. Med. Chem..

[18] Yang C.C., Hu H.S., Wu R.H., Wu S.H., Lee S.J., Jiaang W.T., Chern J.H., Huang Z.S., Wu H.N., Chang C.M. (2014). A novel dengue virus inhibitor, BP13944, discovered by high-throughput screening with dengue virus replicon cells selects for resistance in the viral NS2B/NS3 protease. Antimicrob. Agents Chemother..

[19] Saudi M., Zmurko J., Kaptein S., Rozenski J., Neyts J., Van Aerschot A. (2014). Synthesis and evaluation of imidazole-4,5- and pyrazine-2,3-dicarboxamides targeting dengue and yellow fever virus. Eur. J. Med. Chem..

[20] Behnam M.A.M., Graf D., Bartenschlager R., Zlotos D.P., Klein C.D. (2015). Discovery of Nanomolar Dengue and West Nile Virus Protease Inhibitors Containing a 4-Benzyloxyphenylglycine Residue. J. Med. Chem..

[21] Yokokawa F., Nilar S., Noble C.G., Lim S.P., Rao R., Tania S., Wang G., Lee G., Hunziker J., Karuna R. (2016). Discovery of Potent Non-Nucleoside Inhibitors of Dengue Viral RNA-Dependent RNA Polymerase from a Fragment Hit Using Structure-Based Drug Design. J. Med. Chem..

[22] Bardiot D., Koukni M., Smets W., Carlens G., McNaughton M., Kaptein S., Dallmeier K., Chaltin P., Neyts J., Marchand A. (2018). Discovery of Indole Derivatives as Novel and Potent Dengue Virus Inhibitors. J. Med. Chem..

[23] Millies B., von Hammerstein F., Gellert A., Hammerschmidt S., Barthels F., Göppel U., Immerheiser M., Elgner F., Jung N., Basic M. (2019). Proline-Based Allosteric Inhibitors of Zika and Dengue Virus NS2B/NS3 Proteases. J. Med. Chem..

[24] Xu J., Xie X., Chen H., Zou J., Xue Y., Ye N., Shi P.Y., Zhou J. (2020). Design, synthesis and biological evaluation of spiropyrazolopyridone derivatives as potent dengue virus inhibitors. Bioorg. Med. Chem Lett..

[25] Chen W.C., Simanjuntak Y., Chu L.W., Ping Y.H., Lee Y.L., Lin Y.L., Li W.S. (2020). Benzenesulfonamide Derivatives as Calcium/Calmodulin-Dependent Protein Kinase Inhibitors and Antiviral Agents against Dengue and Zika Virus Infections. J. Med. Chem..

[26] Venkatesham A., Saudi M., Kaptein S., Neyts J., Rozenski J., Froeyen M., Van Aerschot A. (2017). Aminopurine and aminoquinazoline scaffolds for development of potential dengue virus inhibitors. Eur. J. Med. Chem..

[27] Yang Y., Cao L., Gao H., Wu Y., Wang Y., Fang F., Lan T., Lou Z., Rao Y. (2019). Discovery, Optimization, and Target Identification of Novel Potent Broad-Spectrum Antiviral Inhibitors. J. Med. Chem..

[28] Opsenica I., Burnett J.C., Gussio R., Opsenica D., Todorović N., Lanteri C.A., Sciotti R.J., Gettayacamin M., Basilico N., Taramelli D. (2011). A chemotype that inhibits three unrelated pathogenic targets: The botulinum neurotoxin serotype A light chain, *P. falciparum* malaria, and the Ebola filovirus. J. Med. Chem..

[29] Chao B., Tong X.K., Tang W., Li D.W., He P.L., Garcia J.M., Zeng L.M., Gao A.H., Yang L., Li J. (2012). Discovery and optimization of 2,4-diaminoquinazoline derivatives as a new class of potent dengue virus inhibitors. J. Med. Chem..

[30] Vincetti P., Caporuscio F., Kaptein S., Gioiello A., Mancino V., Suzuki Y., Yamamoto N., Crespan E., Lossani A., Maga G. (2015). Discovery of Multitarget Antivirals Acting on Both the Dengue Virus NS5-NS3 Interaction and the Host Src/Fyn Kinases. J. Med. Chem..

[31] Wang Q.Y., Patel S.J., Vangrevelinghe E., Xu H.Y., Rao R., Jaber D., Schul W., Gu F., Heudi O., Ma N.L. (2009). A small-molecule dengue virus entry inhibitor. Antimicrob. Agents Chemother..

[32] Saul S., Pu S.Y., Zuercher W.J., Einav S., Asquith C.R.M. (2020). Potent antiviral activity of novel multi-substituted 4-anilinoquin(az)olines. Bioorg. Med. Chem. Lett..

[33] Saul S., Huang P.T., Einav S., Asquith C.R.M. (2021). Evaluation and identification of 4-anilinoquin(az)olines as potent inhibitors of both dengue virus (DENV) and Venezuelan equine encephalitis virus (VEEV). Bioorg. Med. Chem. Lett..

[34] Davis M.I., Hunt J.P., Herrgard S., Ciceri P., Wodicka L.M., Pallares G., Hocker M., Treiber D.K., Zarrinkar P.P. (2011). Comprehensive analysis of kinase inhibitor selectivity. Nat. Biotechnol..

[35] Fabian M.A., Biggs W.H., Treiber D.K., Atteridge C.E., Azimioara M.D., Benedetti M.G., Carter T.A., Ciceri P., Edeen P.T., Floyd M. (2005). A small molecule-kinase interaction map for clinical kinase inhibitors. Nat. Biotechnol..

[36] Klaeger S., Heinzlmeir S., Wilhelm M., Polzer H., Vick B., Koenig P.-A., Reinecke M., Ruprecht B., Petzoldt S., Meng C. (2017). The target landscape of clinical kinase drugs. Science.

[37] Attwood M.M., Fabbro D., Sokolov A.V., Knapp S., Schiöth H.B. (2021). Trends in kinase drug discovery: Targets, indications and inhibitor design. Nat. Rev. Drug Discov..

[38] Asquith C.R.M., Laitinen T., Bennett J.M., Godoi P.H., East M.P., Tizzard G.H., Graves L.M., Johnson G.L., Dornsife R.E., Wells C.I. (2018). Identification and optimization of 4-anilinoquinolines as inhibitors of cyclin G associated kinase. ChemMedChem.

[39] Asquith C.R.M., Berger B.T., Wan J., Bennett J.M., Capuzzi S.J., Crona D.J., Drewry D.H., East M.P., Elkins J.M., Fedorov O. (2019). SGC-GAK-1: A Chemical Probe for Cyclin G Associated Kinase (GAK). J. Med. Chem..

[40] Asquith C.R.M., Naegeli K.M., East M.P., Laitinen T., Havener T.M., Wells C.I., Johnson G.L., Drewry D.H., Zuercher W.J., Morris D.C. (2019). Design of a Cyclin G Associated Kinase (GAK)/Epidermal Growth Factor Receptor (EGFR) Inhibitor Set to Interrogate the Relationship of EGFR and GAK in Chordoma. J. Med. Chem..

[41] Asquith C.R.M., Treiber D.K., Zuercher W.J. (2019). Utilizing comprehensive and mini-kinome panels to optimize the selectivity of quinoline inhibitors for cyclin G associated kinase (GAK). Bioorg. Med. Chem. Lett..

[42] Asquith C.R.M., Bennett J.M., Su L., Laitinen T., Elkins J.M., Pickett J.E., Wells C.I., Li Z., Willson T.M., Zuercher W.J. (2019). Towards the Development of an In vivo Chemical Probe for Cyclin G Associated Kinase (GAK). Molecules.

[43] Asquith C.R.M., Fleck N., Torrice C.D., Crona D.J., Grundner C., Zuercher W.J. (2019). Anti-tubercular activity of novel 4-anilinoquinolines and 4-anilinoquinazolines. Bioorg. Med. Chem. Lett..

[44] Asquith C.R.M., Maffuid K.A., Laitinen T., Torrice C.D., Tizzard G.J., Crona D.J., Zuercher W.J. (2019). Targeting an EGFR Water Network with 4-Anilinoquin(az)oline Inhibitors for Chordoma. ChemMedChem.

[45] Carabajal M.A., Asquith C.R.M., Laitinen T., Tizzard G.J., Yim L., Rial A., Chabalgoity J.A., Zuercher W.J., García Véscovi E. (2019). Quinazoline-Based Antivirulence Compounds Selectively Target Salmonella PhoP/PhoQ Signal Transduction System. Antimicrob. Agents Chemother..

[46] Asquith C.R.M., Laitinen T., Wells C.I., Tizzard G.J., Zuercher W.J. (2020). New Insights into 4-Anilinoquinazolines as Inhibitors of Cardiac Troponin I-Interacting Kinase (TNNi3K). Molecules.

[47] Asquith C.R.M., Laitinen T., Bennett J.M., Wells C.I., Elkins J.M., Zuercher W.J., Tizzard G.J., Poso A. (2020). Design and Analysis of the 4-Anilinoquin(az)oline Kinase Inhibition Profiles of GAK/SLK/STK10 Using Quantitative Structure-Activity Relationships. ChemMedChem.

[48] Asquith C.R.M., Tizzard G.J., Bennett J.M., Wells C.I., Elkins J.M., Willson T.M., Poso A., Laitinen T. (2020). Targeting the Water Network in Cyclin G-Associated Kinase (GAK) with 4-Anilino-quin(az)oline Inhibitors. ChemMedChem.

[49] Savoie P.R., Welch J.T. (2015). Preparation and utility of organic pentafluorosulfanyl-containing compounds. Chem. Rev..

[50] Ammerman N.C., Beier-Sexton M., Azad A.F. (2008). Growth and maintenance of Vero cell lines. Curr. Protoc. Microbiol..

[51] Munoz L. (2017). Non-kinase targets of protein kinase inhibitors. Nat. Rev. Drug Discov..

[52] Haese N.N., May N.A., Taft-Benz S., Moukha-Chafiq O., Madadi N., Zhang S., Karyakarte S.D., Rodzinak K.J., Nguyen T.H., Denton M. (2021). Identification of Quinolinones as Antivirals against Venezuelan Equine Encephalitis Virus. Antimicrob Agents Chemother..

[53] Chung D.H., Jonsson C.B., Tower N.A., Chu Y.K., Sahin E., Golden J.E., Noah J.W., Schroeder C.E., Sotsky J.B., Sosa M.I. (2014). Discovery of a novel compound with anti-venezuelan equine encephalitis virus activity that targets the nonstructural protein 2. PLoS Pathog..

[54] Persoons L., Vanderlinden E., Vangeel L., Wang X., Do N.D.T., Foo S.C., Leyssen P., Neyts J., Jochmans D., Schols D. (2021). Broad spectrum anti-coronavirus activity of a series of anti-malaria quinoline analogues. Antiviral Res..

[55] Xie X., Gayen S., Kang C., Yuan Z., Shi P.-Y. (2013). Membrane topology and function of dengue virus NS2A protein. J. Virol..

[56] Zou G., Xu H.Y., Qing M., Wang Q.Y., Shi P.Y. (2011). Development and characterization of a stable luciferase dengue virus for high-throughput screening. Antiviral Res..

[57] Sun C., Gardner C.L., Watson A.M., Ryman K.D., Klimstra W.B. (2014). Stable, high-level expression of reporter proteins from improved alphavirus expression vectors to track replication and dissemination during encephalitic and arthritogenic disease. J. Virol..

