# Supplementary material for: Optimization of 4-Anilinoquinolines as Dengue Virus Inhibitors
Source: Molecules. 2021 Dec 3;26(23):7338. doi: 10.3390/molecules26237338 (PMC8659069; doi:10.3390/molecules26237338)
Supplement: Supplementary file 1 [file molecules-26-07338-s001.zip › molecules-1447606-supplementary.pdf]

Supplementary Materials

Optimization of 4-Anilinoquinolines as Dengue Virus Inhibitors

Pei-Tzu Huang [1,2,†], Sirle Saul [1,2,†], Shirit Einav [1,2,*] and Christopher R. M. Asquith [3,4,*]

[1] Department of Medicine, Division of Infectious Diseases and Geographic Medicine, Stanford University School of Medicine, Stanford, CA 94305, USA. pthuang@stanford.edu (P.-T.H.); sauls@stanford.edu (S.S.)
[2] Department of Microbiology and Immunology, Stanford University School of Medicine, Stanford, CA 94305, USA
[3] Department of Pharmacology, School of Medicine, University of North Carolina at Chapel Hill, Chapel Hill, NC 27599, USA
[4] Structural Genomics Consortium, UNC Eshelman School of Pharmacy, University of North Carolina at Chapel Hill, Chapel Hill, NC 27599, USA
† These authors contributed equally to this work.
* Correspondence: seinav@stanford.edu (S.E.); chris.asquith@unc.edu (C.R.M.A.); Tel.: +1-650-723-8656 (S.E.); Tel.: +1-919-491-3177 (C.R.M.A.)

Representative Supporting information

See main manuscript references: 38, 42 and 48 for complete details.

Chemistry: All reactions were performed using flame-dried round-bottomed flasks or reaction vessels. Where appropriate, reactions were carried out under an inert atmosphere of nitrogen with dry solvents, unless otherwise stated. Yields refer to chromatographically and spectroscopically pure isolated yields. Reagents were purchased at the highest commercial quality and used without further purification. Reactions were monitored by thin-layer chromatography carried out on 0.25 mm E. Merck silica gel plates ($60_{F\text{-}254}$) using ultraviolet light as the visualizing agent. NMR spectra were recorded on a Varian Inova 400 spectrometer (Varian, Palo Alto, CA, USA) and were calibrated using residual undeuterated solvent as an internal reference ($CDCl_3$: 1H-NMR = 7.26, ^{13}C-NMR = 77.16). The following abbreviations or combinations thereof have been used to explain the multiplicities observed: s = singlet, d = doublet, t = triplet, q = quartet, m = multiplet, br = broad. Liquid chromatography (LC) and high-resolution mass spectra (HRMS) were recorded on a ThermoFisher hybrid LTQ FT (ICR 7T) (ThermoFisher, Waltham, MA, USA). Melting points were consistent with previous reports [38, 42, 48].

Mass Spectrometry Method: Samples were analyzed with a ThermoFisher Q Exactive HF-X (ThermoFisher, Bremen, Germany) mass spectrometer coupled with a Waters Acquity H-class liquid chromatograph system. Samples were introduced via a heated electrospray source (HESI) at a flow rate of 0.6 mL/min. Electrospray source conditions were set as: spray voltage 3.0 kV, sheath gas (nitrogen) 60 arb, auxiliary gas (nitrogen) 20 arb, sweep gas (nitrogen) 0 arb, nebulizer temperature 375 degrees C, capillary temperature 380 degrees C, RF funnel 45 V. The mass range was set to 150-2000 m/z. All measurements were recorded at a resolution setting of 120,000.

Separations were conducted on a Waters Acquity UPLC BEH C18 column (2.1 x 50 mM, 1.7 μM particle size). LC conditions were set at 100 % water with 0.1 % formic acid (A) ramped linearly over 9.8 mins to 95 % acetonitrile with 0.1 % formic acid (B) and held until 10.2 mins. At 10.21 mins the gradient was switched back to 100% A and allowed to re-equilibrate until 11.25 mins. Injection volume for all samples was 3 μL.

Xcalibur (ThermoFisher, Breman, Germany) was used to analyze the data. Solutions were analyzed at 0.1 mg/mL or less based on responsiveness to the ESI mechanism. Molecular formula assignments were determined with Molecular Formula Calculator (v 1.2.3). All observed species were singly charged, as verified by unit m/z separation between mass spectral peaks corresponding to the ^{12}C and $^{13}C^{12}C_{c-1}$ isotope for each elemental composition.

General procedure for the synthesis of 4-anilinoquin(az)olines: 4-chloroquin(az)oline derivative (1.0 eq.), aniline derivative (1.1 eq.), were suspended in ethanol (10 mL) and refluxed for 18 h. The crude mixture was purified by flash chromatography using EtOAc:hexane followed by 1-5 % methanol in EtOAc; After solvent removal under reduced pressure, the product was obtained as a free following solid or recrystallized from ethanol/water. Compounds **4**-**31** were synthesized as previous described [38, 48], **32**-**51** were synthesized as previous described [42]. Representative supporting information provided.

N-(3-Methoxyphenyl)-6-(trifluoromethyl)quinolin-4-amine (**4**) was obtained as a light yellow solid (258 mg, 94 %); m.p. 135-137 °C; ^{1}H NMR (400 MHz, DMSO-d$_6$) δ 11.24 (s, 1H), 9.34 (s, 1H), 8.58 (d, J = 6.8 Hz, 1H), 8.28 (q, J = 8.9 Hz, 2H), 7.48 (t, J = 8.3 Hz, 1H), 7.07 (s, 2H), 6.96 (dd, J = 26.3, 7.3 Hz, 2H), 3.81 (s, 3H); ^{13}C NMR (101 MHz, DMSO-d$_6$) δ 160.6, 154.8, 145.4, 141.6, 138.6, 131.0, 129.1 (d, J = 3.4 Hz), 126.7 (q, J = 32.8 Hz, CF_3), 125.4, 123.2, 122.7 (d, J = 3.9 Hz), 117.2, 117.2, 113.2, 110.9, 101.6, 55.6; HRMS-ESI (m/z): [M+H]$^+$ calcd for $C_{17}H_{13}F_3N_2O$ - 319.1058; found 319.1048; T_r = 4.08 min, purity >95 %.

6-bromo-*N*-(3-methoxyphenyl)quinolin-4-amine (**11**) was obtained as a beige solid (151 mg, 0.458 mmol, 74 %). MP 188-190 °C; $_1$H NMR (400 MHz, DMSO-*d*$_6$) δ 11.07 (s, 1H), 9.16 (d, *J* = 2.0 Hz, 1H), 8.52 (d, *J* = 7.0 Hz, 1H), 8.17 (dd, *J* = 9.0, 2.0 Hz, 1H), 8.08 (d, *J* = 9.0 Hz, 1H), 7.48 (t, *J* = 8.4 Hz, 1H), 7.31 – 7.03 (m, 2H), 7.00 (ddd, *J* = 8.3, 2.4, 1.0 Hz, 1H), 6.89 (d, *J* = 6.9 Hz, 1H), 3.81 (s, 3H). $_{13}$C NMR (100 MHz, DMSO-*d*$_6$) δ $_{13}$C NMR (101 MHz, DMSO-*d*$_6$) δ 160.3, 153.9, 143.0, 138.2, 137.4, 136.6, 130.8, 126.2, 122.5, 119.8, 118.6, 117.1, 113.1, 110.9, 100.7, 55.4. HRMS *m/z* [M+H]$_+$ calcd for $C_{16}H_{14}N_2BrO$: 329.0286, found 329.0289, LC t_R = 3.63 min, > 98 % Purity.

3-((6-bromoquinolin-4-yl)amino)phenol (**12**) was obtained as a yellow solid (138 mg, 0.437 mmol, 71 %). MP >300 °C; $_1$H NMR (400 MHz, DMSO-*d*$_6$) δ 11.00 (s, 1H), 10.03 (s, 1H), 9.14 (d, *J* = 2.0 Hz, 1H), 8.51 (d, *J* = 7.0 Hz, 1H), 8.15 (dd, *J* = 9.0, 2.0 Hz, 1H), 8.06 (d, *J* = 9.0 Hz, 1H), 7.42 – 7.29 (m, 1H), 7.10 – 6.66 (m, 4H). $_{13}$C NMR (100 MHz, DMSO-*d*$_6$) δ 159.2, 154.4, 143.3, 138.3, 137.7, 137.0, 131.1, 126.6, 122.8, 120.2, 119.0, 115.9, 115.2, 112.5, 101.0. HRMS *m/z* [M+H]$_+$ calcd for $C_{15}H_{11}BrN_2O$: 315.0132, found 315.0124, LC t_R = 3.01 min, > 98 % Purity.

6-Bromo-N-(5-fluoro-1H-indazol-6-yl)quinolin-4-amine (**50**) was obtained as a beige solid (188 mg, 0.526 mmol, 85%). MP >300 °C; ^{1}H NMR (400 MHz, DMSO- *d*$_6$) δ 13.51 (s, 1H), 11.24 (s, 1H), 9.23 (d, J = 2.0 Hz, 1H), 8.56 (d, J = 6.9 Hz, 1H), 8.49 – 8.13 (m, 2H), 8.11 (d, J = 9.0 Hz, 1H), 7.88 (d, J = 10.3 Hz, 1H), 7.80 (dd, J = 6.3, 1.1 Hz, 1H), 6.61 (dd, J = 6.9, 2.5 Hz, 1H). ^{13}C NMR (100 MHz, DMSO- *d*$_6$) δ 154.8, 151.9 (d, J = 240.3 Hz), 143.3, 137.3, 136.7, 136.5, 134.4 – 133.0 (m, 1C), 126.2, 123.9 (d, J = 17.7 Hz), 122.6, 121.6 (d, J = 9.5 Hz), 120.1, 118.4, 109.8, 106.5 (d, J = 22.1 Hz), 101.2. HRMS m/z [M+H]$^+$ calcd for $C_{16}H_{11}N_4FBr$: 357.0151, found 357.0150, LC t_R = 3.43 min, > 98% Purity.

6-[(6-Bromoquinolin-4-yl)amino]-2,3-dihydro-1H-isoindol-1-one (**52**) was obtained as a yellow solid (160 mg, 0.452 mmol, 73%). MP >300 °C; ^{1}H NMR (400 MHz, DMSO-*d*$_6$) δ 11.19 (s, 1H), 9.18 (d, *J* = 2.0 Hz, 1H), 8.77 (s, 1H), 8.54 (d, *J* = 6.9 Hz, 1H), 8.16 (dd, *J* = 9.0, 2.0 Hz, 1H), 8.07 (d, *J* = 9.0 Hz, 1H), 7.95 – 7.72 (m, 2H), 7.70 (dd, *J* = 8.0, 2.0 Hz, 1H), 6.85 (d, *J* = 6.9 Hz, 1H), 4.45 (s, 2H). ^{13}C NMR (100 MHz, DMSO-*d*$_6$) δ 169.0, 154.0, 143.3, 142.9, 137.5, 136.9, 136.5, 134.3, 128.4, 126.2, 125.4, 122.7, 119.9, 119.4, 118.8, 100.5, 45.0. HRMS *m/z* [M+H]$^+$ calcd for $C_{17}H_{13}N_3OBr$: 354.0242, found 354.0240, LC t_R = 3.46 min, > 98% Purity.

N-(3-Methoxyphenyl)-6-(trifluoromethyl)quinolin-4-amine (**4**)

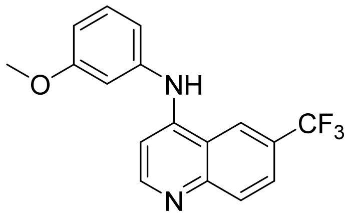

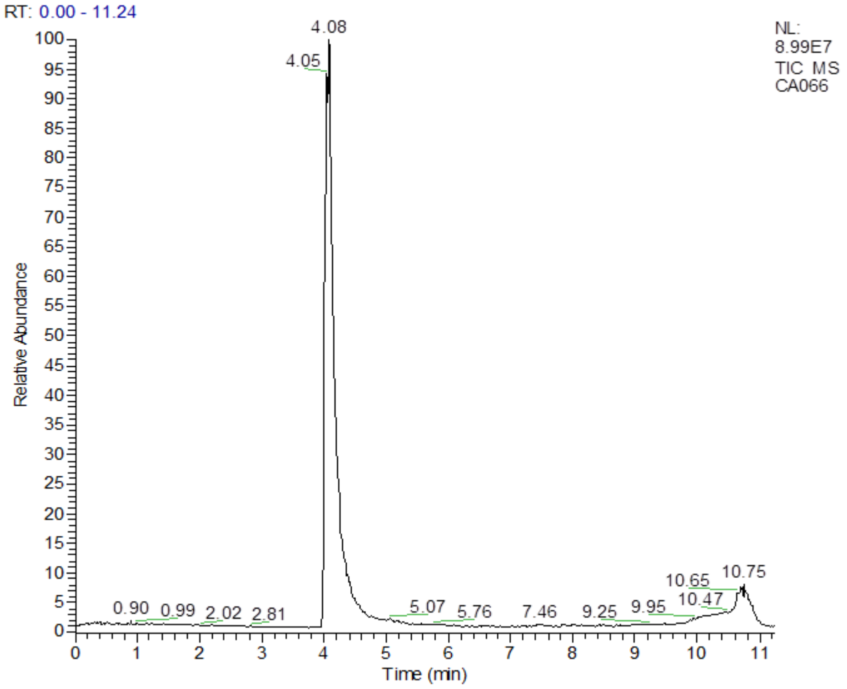

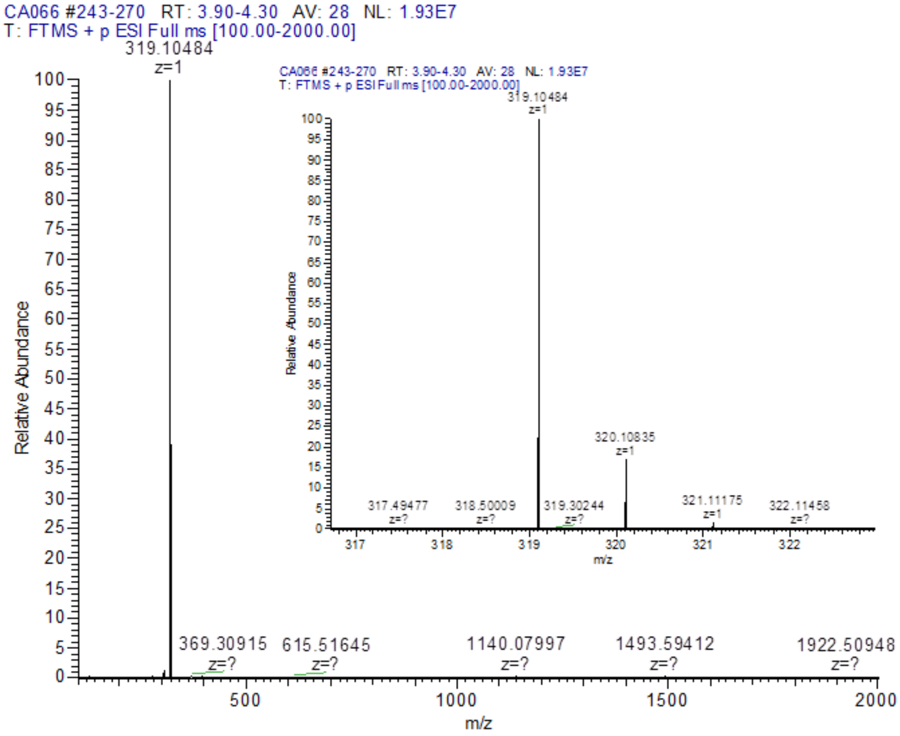

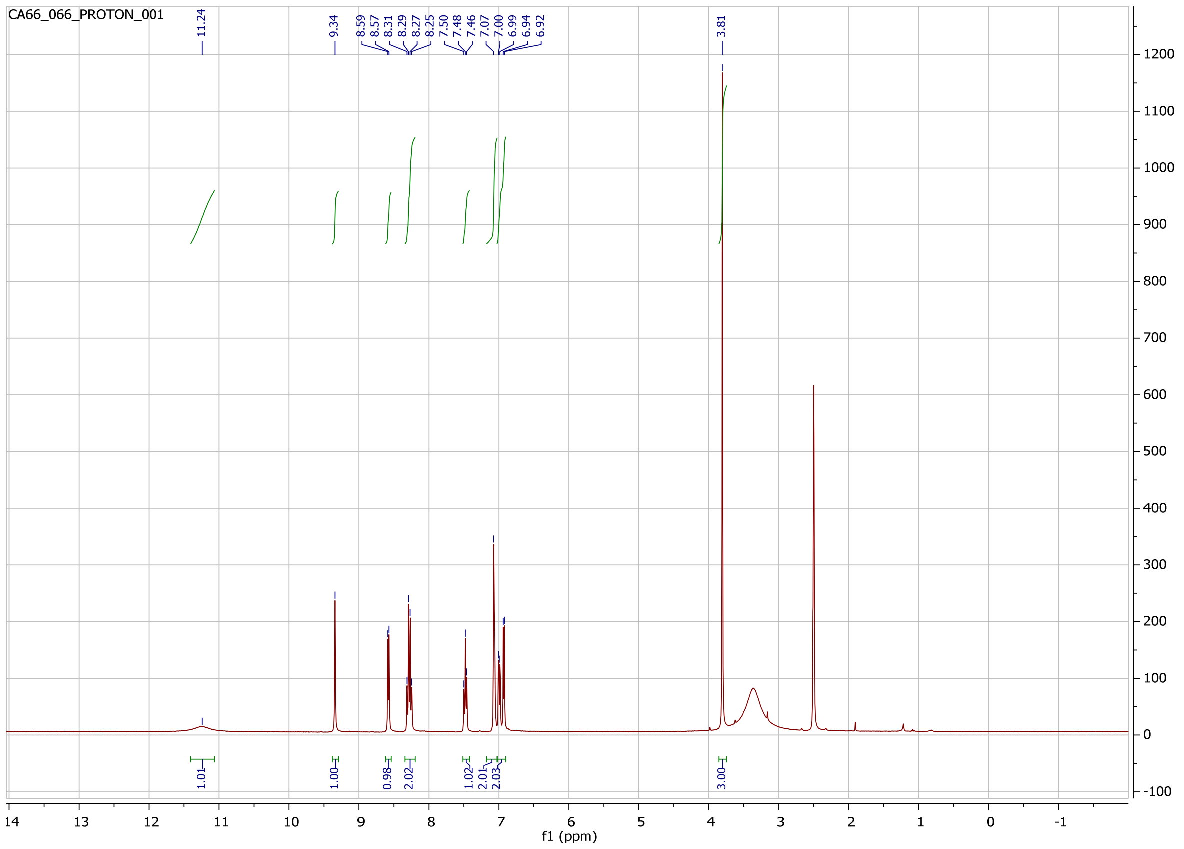
CA66_066_PROTON_001
11.24
9.34
8.59
8.57
8.31
8.29
8.27
8.25
7.50
7.48
7.46
7.07
7.00
6.99
6.94
6.92
3.81
1.01
1.00
0.98
2.02
1.02
2.01
2.03
3.00
f1 (ppm)

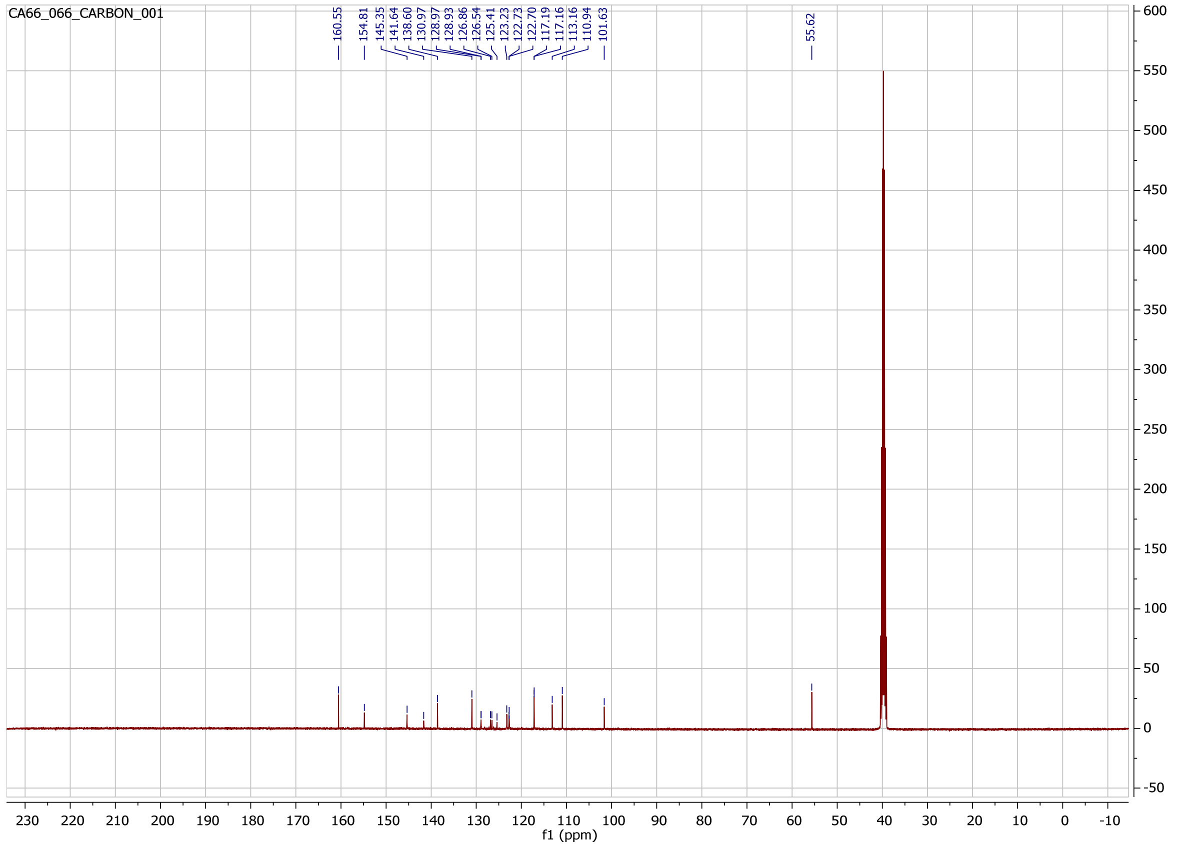
CA66_066_CARBON_001
160.55
154.81
145.35
141.64
138.60
130.97
128.97
128.93
126.86
126.54
125.41
123.23
122.73
122.70
117.19
117.16
113.16
110.94
101.63
55.62
f1 (ppm)

6-bromo-*N*-(3-methoxyphenyl)quinolin-4-amine (**11**)

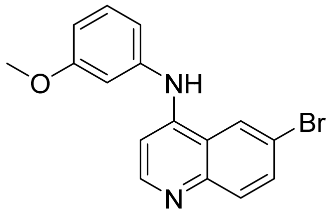

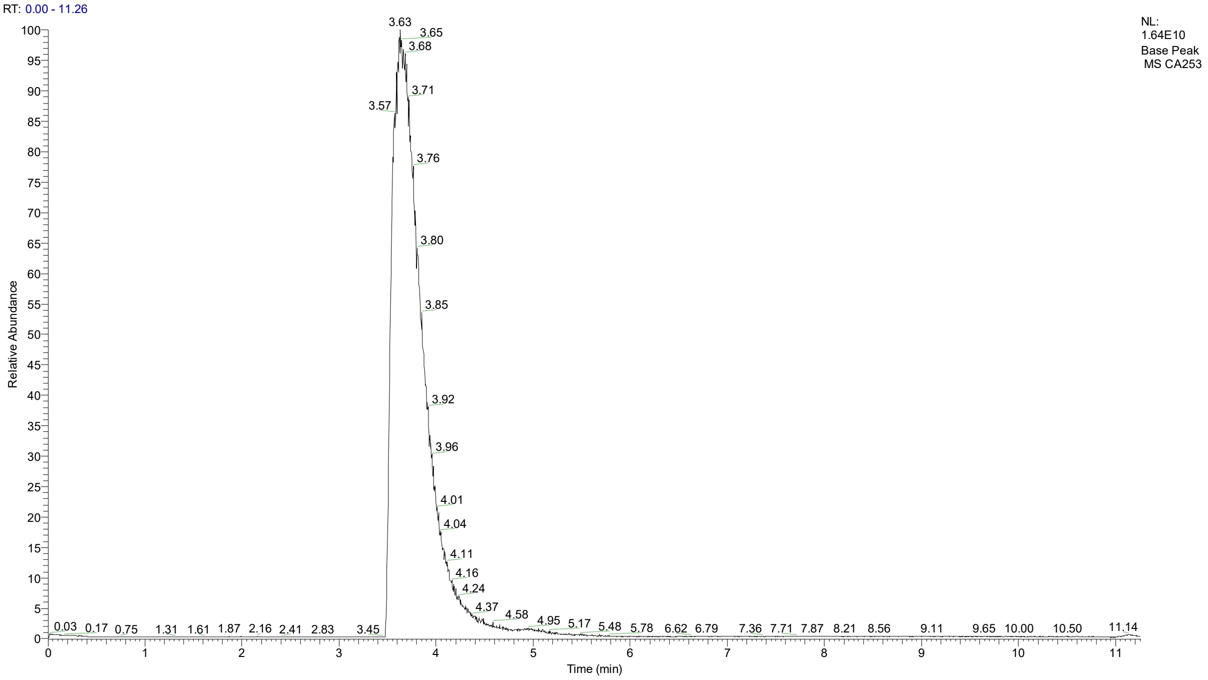

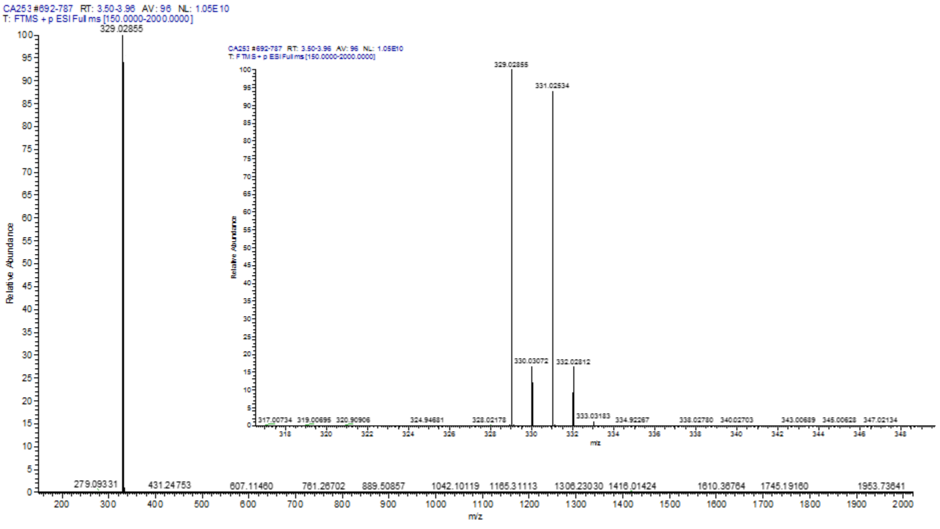

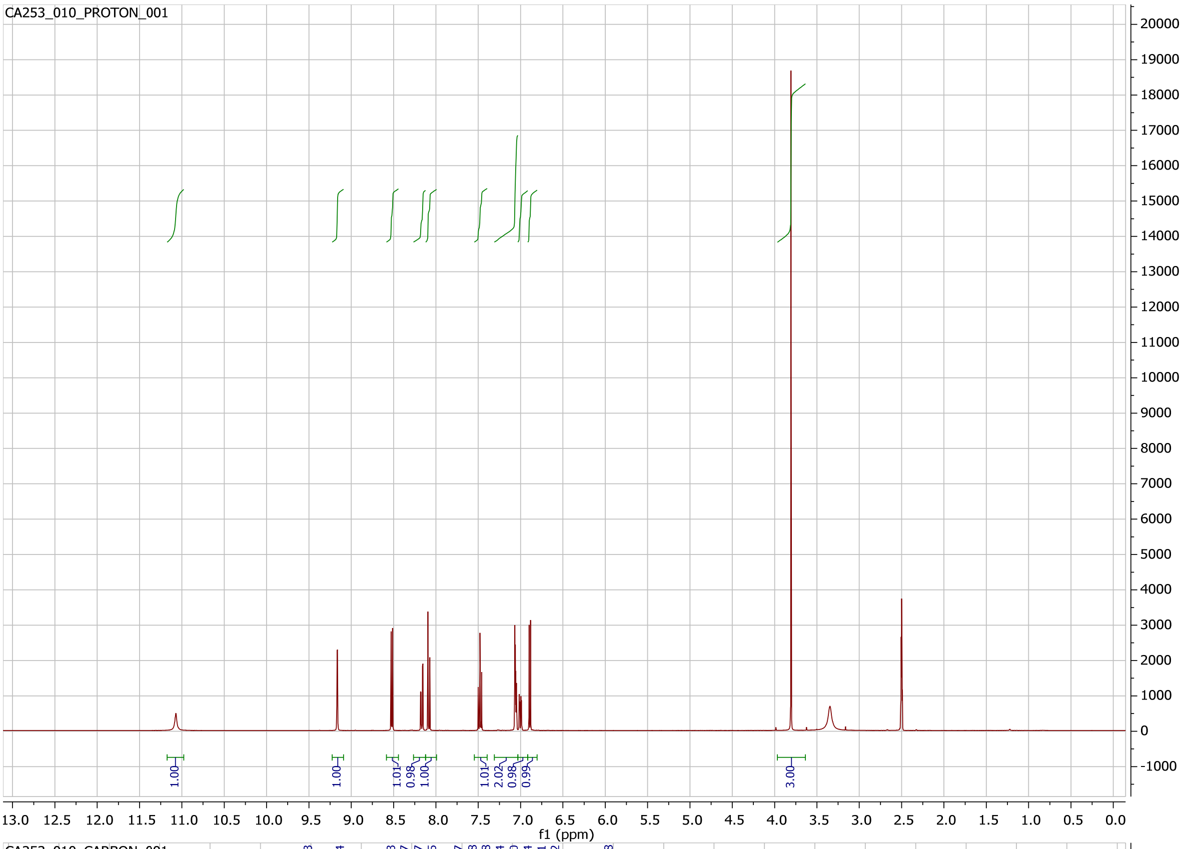
CA253_010_PROTON_001
1.00
1.00
1.01
0.98
1.00
1.01
2.02
0.98
0.99
3.00
f1 (ppm)

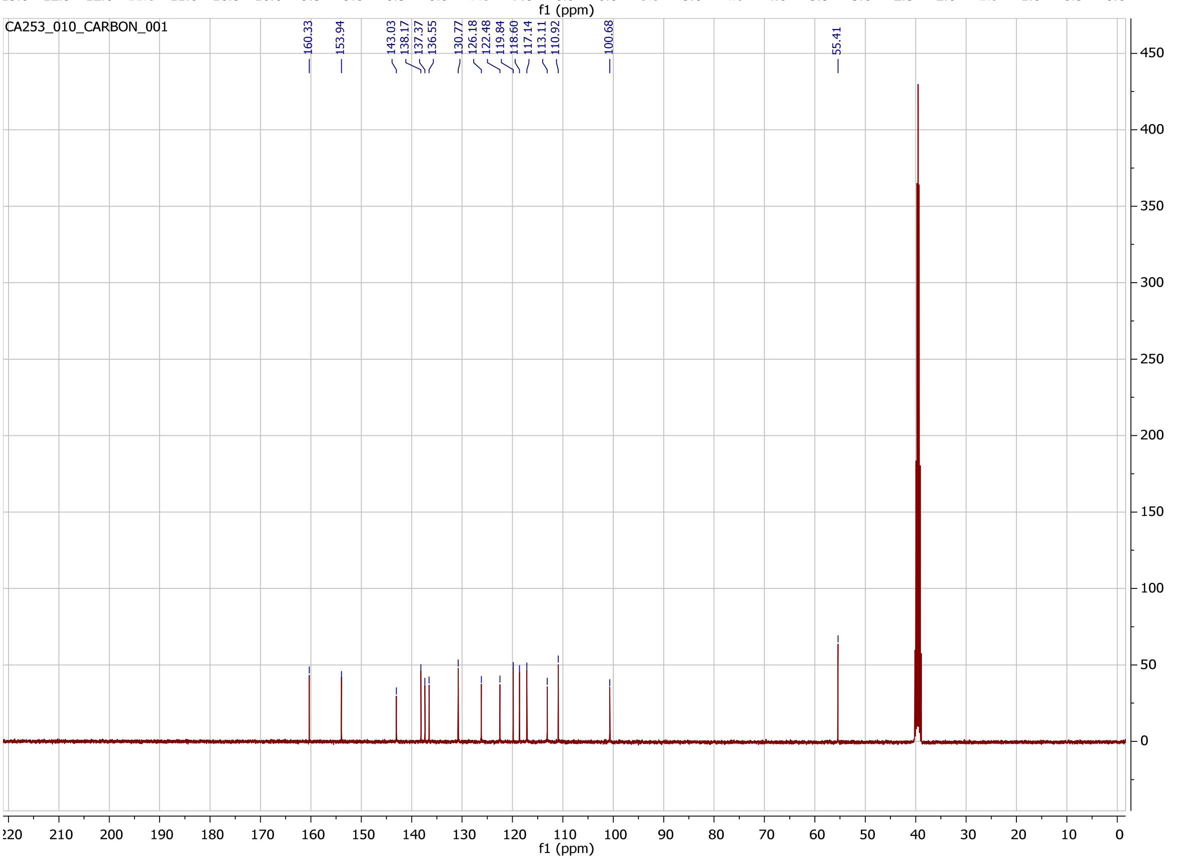
CA253_010_CARBON_001
160.33
153.94
143.03
138.17
137.37
136.55
130.77
126.18
122.48
119.84
118.60
117.14
113.11
110.92
100.68
55.41
f1 (ppm)

3-((6-bromoquinolin-4-yl)amino)phenol (**12**)

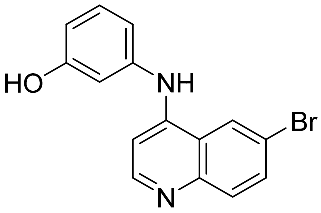

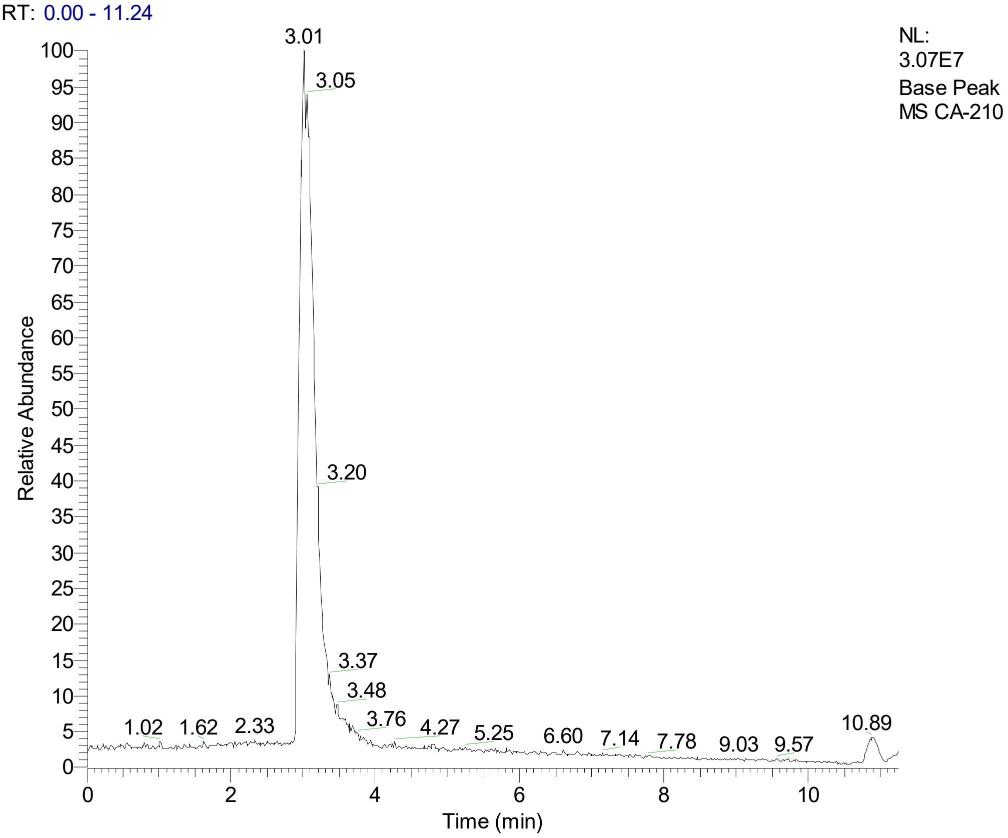

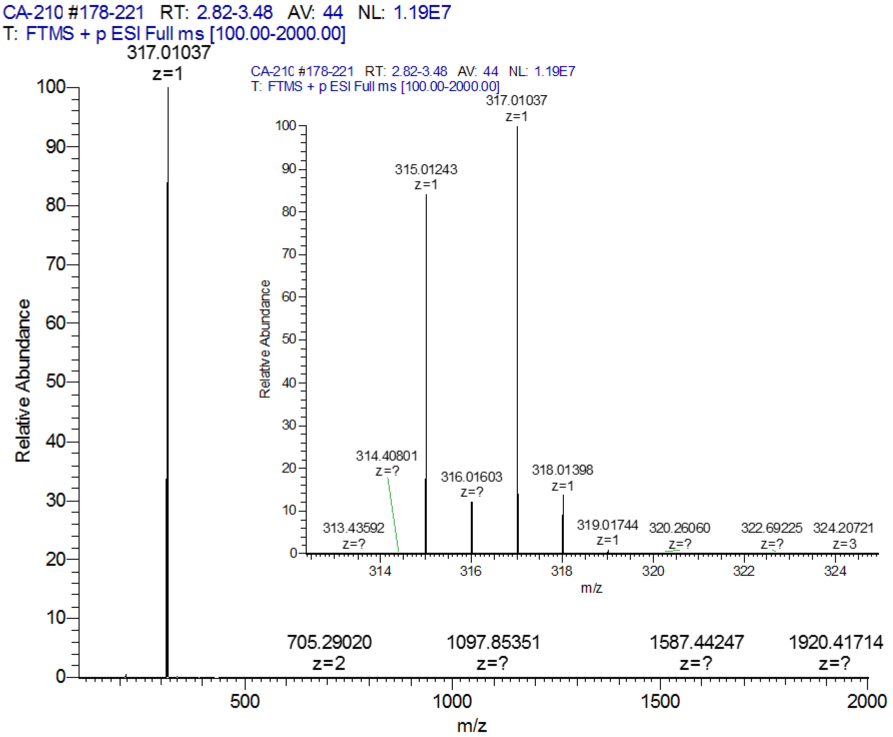

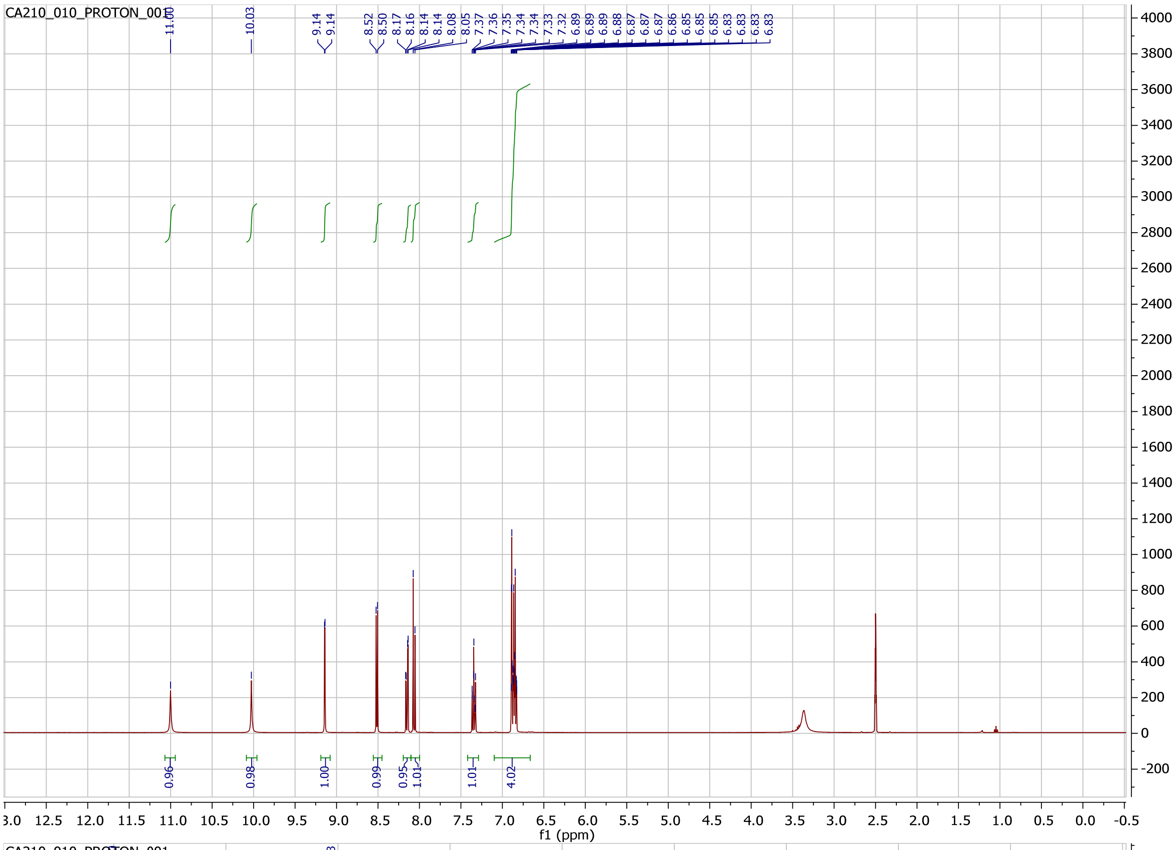
CA210_010_PROTON_001
11.00
10.03
9.14
9.14
8.52
8.50
8.17
8.16
8.14
8.14
8.08
8.05
7.37
7.36
7.35
7.34
7.34
7.33
7.32
6.89
6.89
6.88
6.87
6.87
6.87
6.86
6.85
6.85
6.85
6.83
6.83
6.83
6.83
0.96
0.98
1.00
0.99
0.95
1.01
1.01
4.02
f1 (ppm)

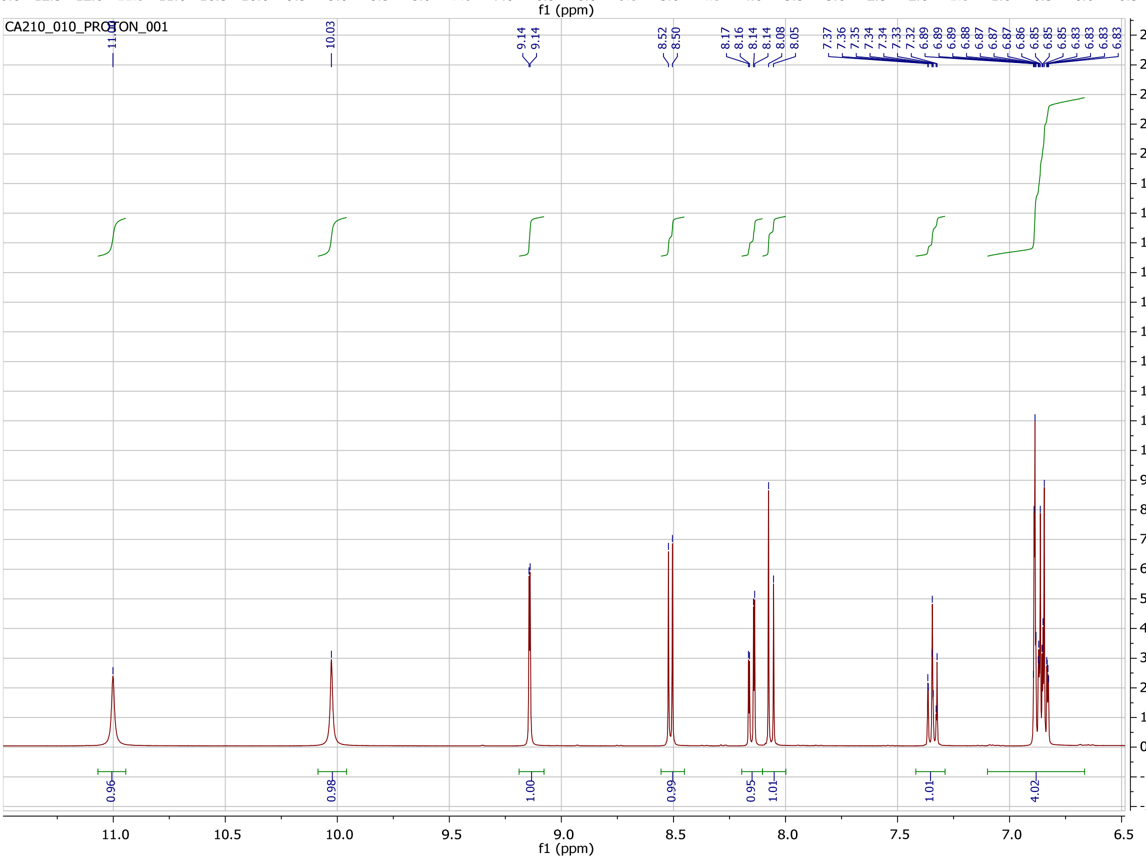
CA210_010_PROTON_001
11.00
10.03
9.14
9.14
8.52
8.50
8.17
8.16
8.14
8.14
8.08
8.05
7.37
7.36
7.35
7.34
7.34
7.33
7.32
6.89
6.89
6.89
6.88
6.87
6.87
6.87
6.86
6.85
6.85
6.85
6.83
6.83
6.83
6.83
0.96
0.98
1.00
0.99
0.95
1.01
1.01
4.02
f1 (ppm)

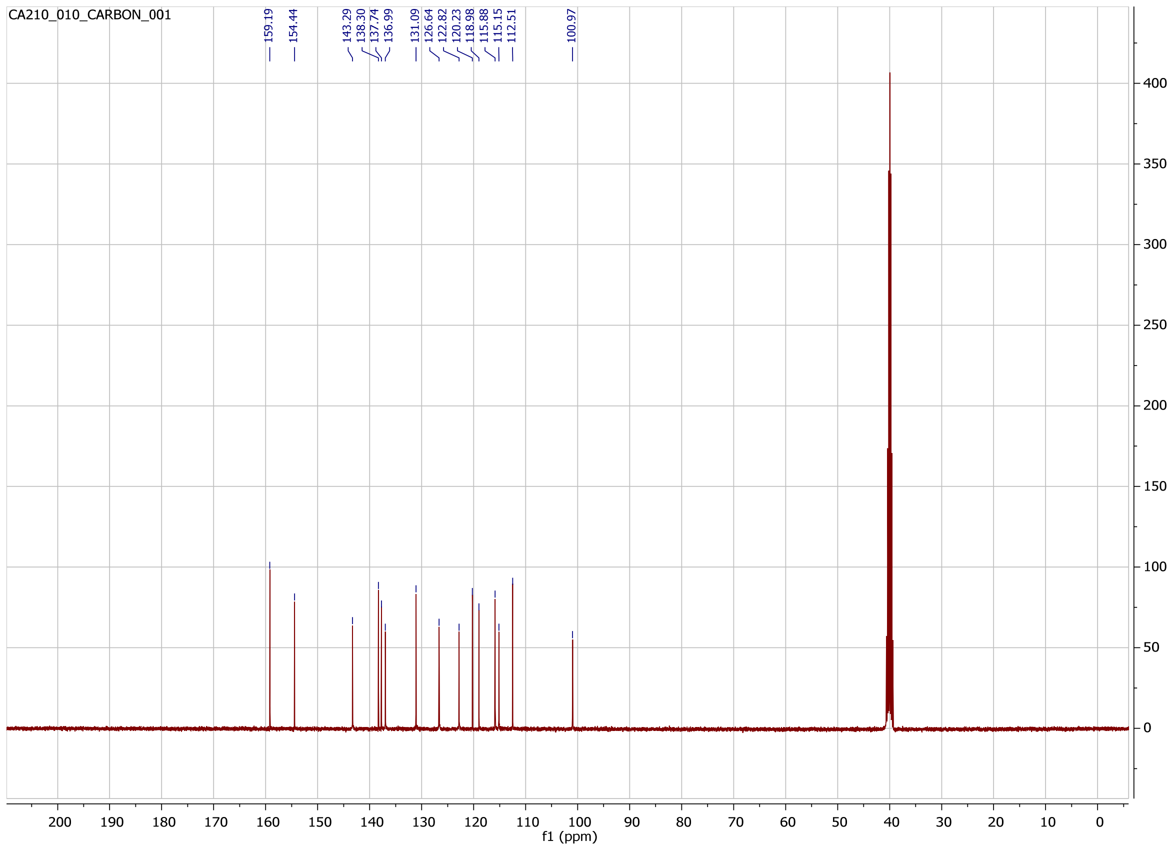
CA210_010_CARBON_001
159.19
154.44
143.29
138.30
137.74
136.99
131.09
126.64
122.82
120.23
118.98
115.88
115.15
112.51
100.97
f1 (ppm)

6-bromo-*N*-(5-fluoro-1*H*-indazol-6-yl)quinolin-4-amine (**50**)

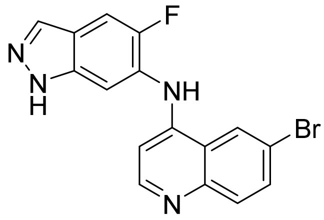

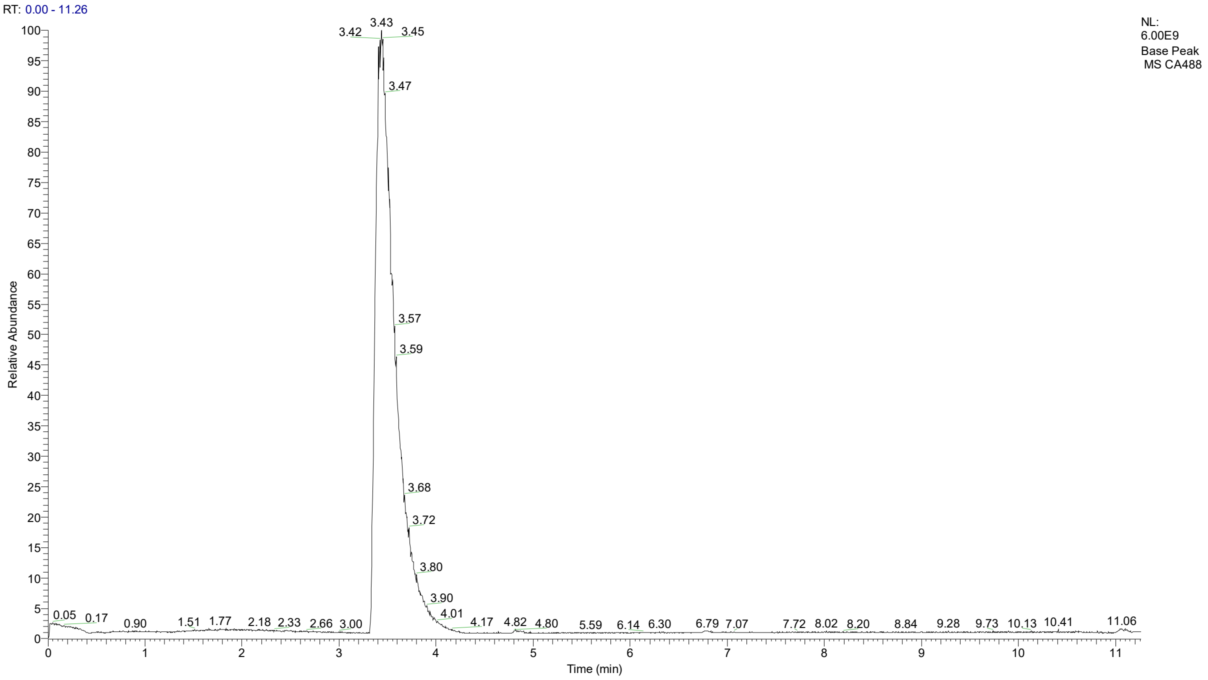

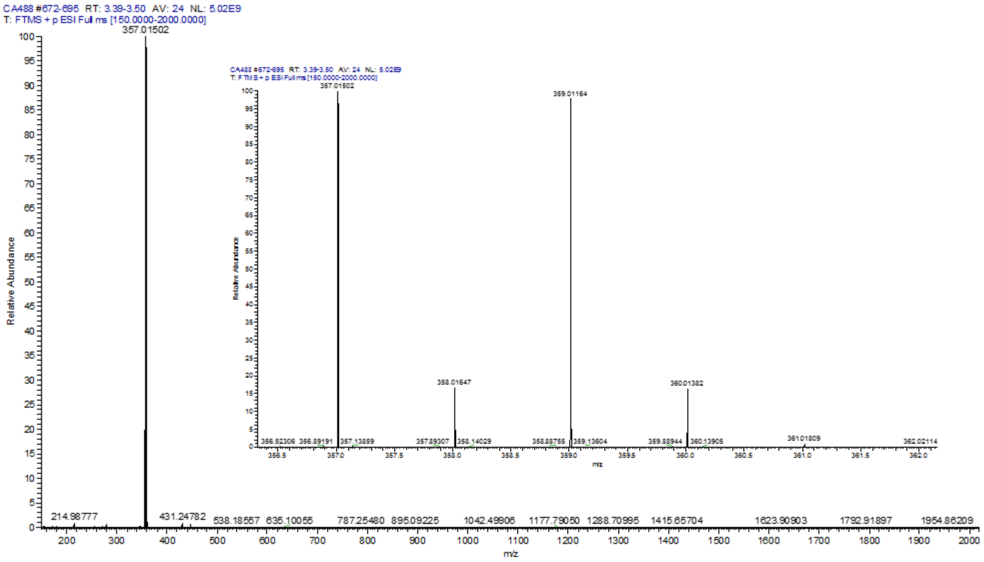

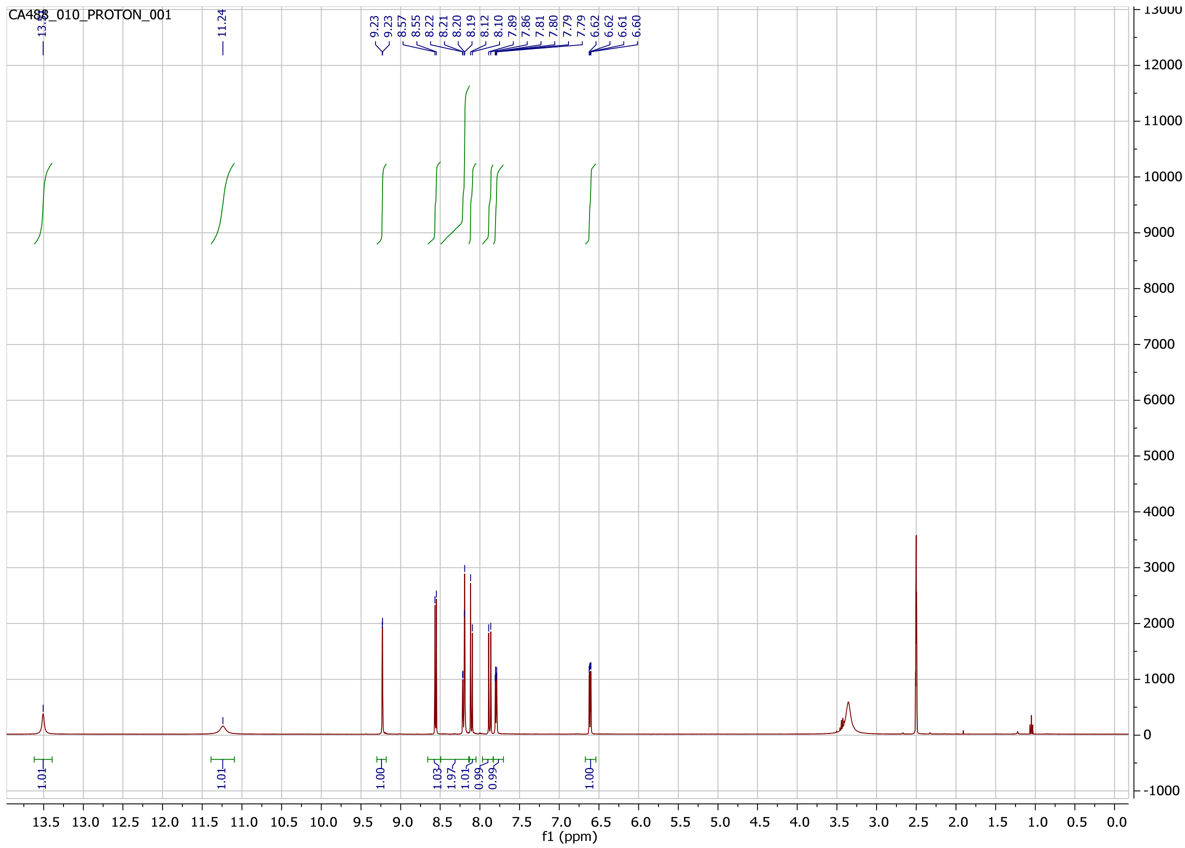
CA488_010_PROTON_001
13.50
11.24
9.23
9.23
8.57
8.55
8.22
8.21
8.20
8.19
8.12
8.10
7.89
7.86
7.81
7.80
7.79
7.79
6.62
6.62
6.61
6.60
1.01
1.01
1.00
1.03
1.97
1.01
0.99
0.99
1.00
f1 (ppm)

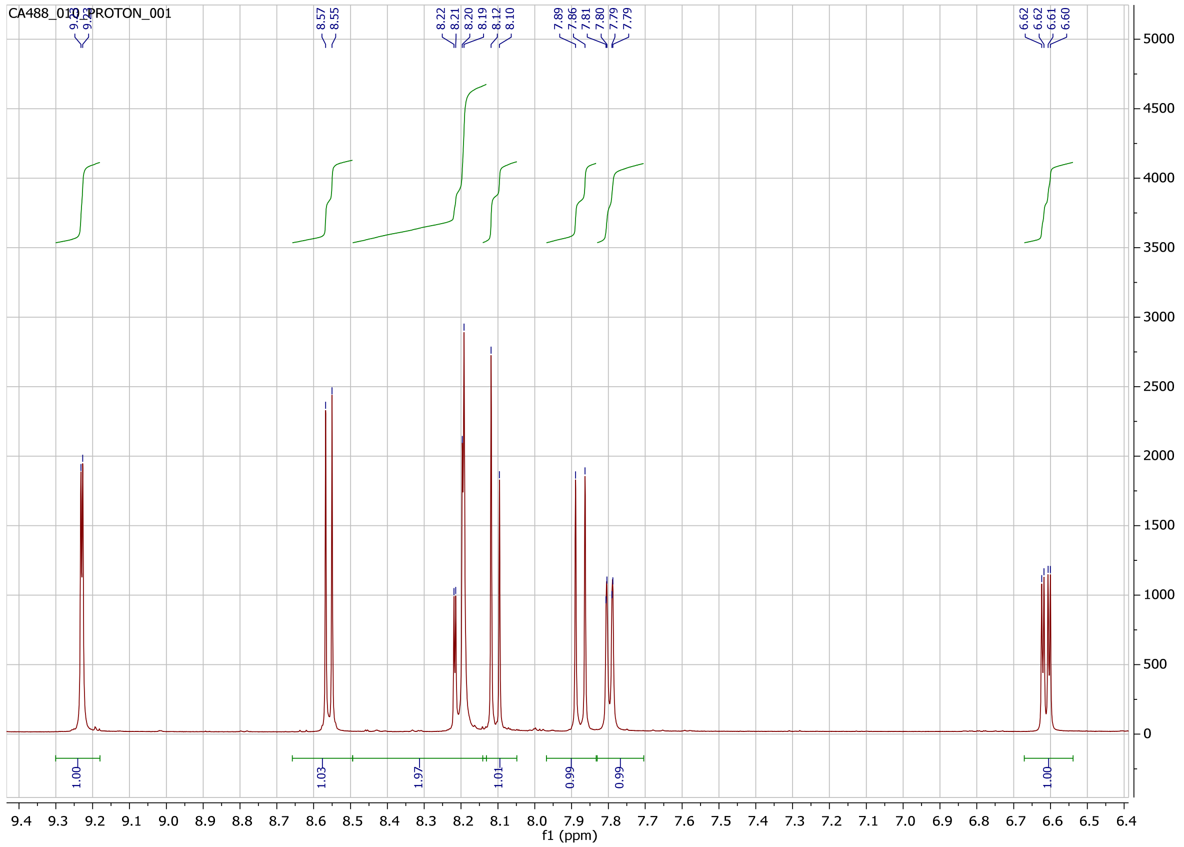
CA488_010_PROTON_001
8.57
8.55
8.22
8.21
8.20
8.19
8.12
8.10
7.89
7.86
7.81
7.80
7.79
7.79
6.62
6.62
6.61
6.60
1.00
1.03
1.97
1.01
0.99
0.99
1.00
f1 (ppm)

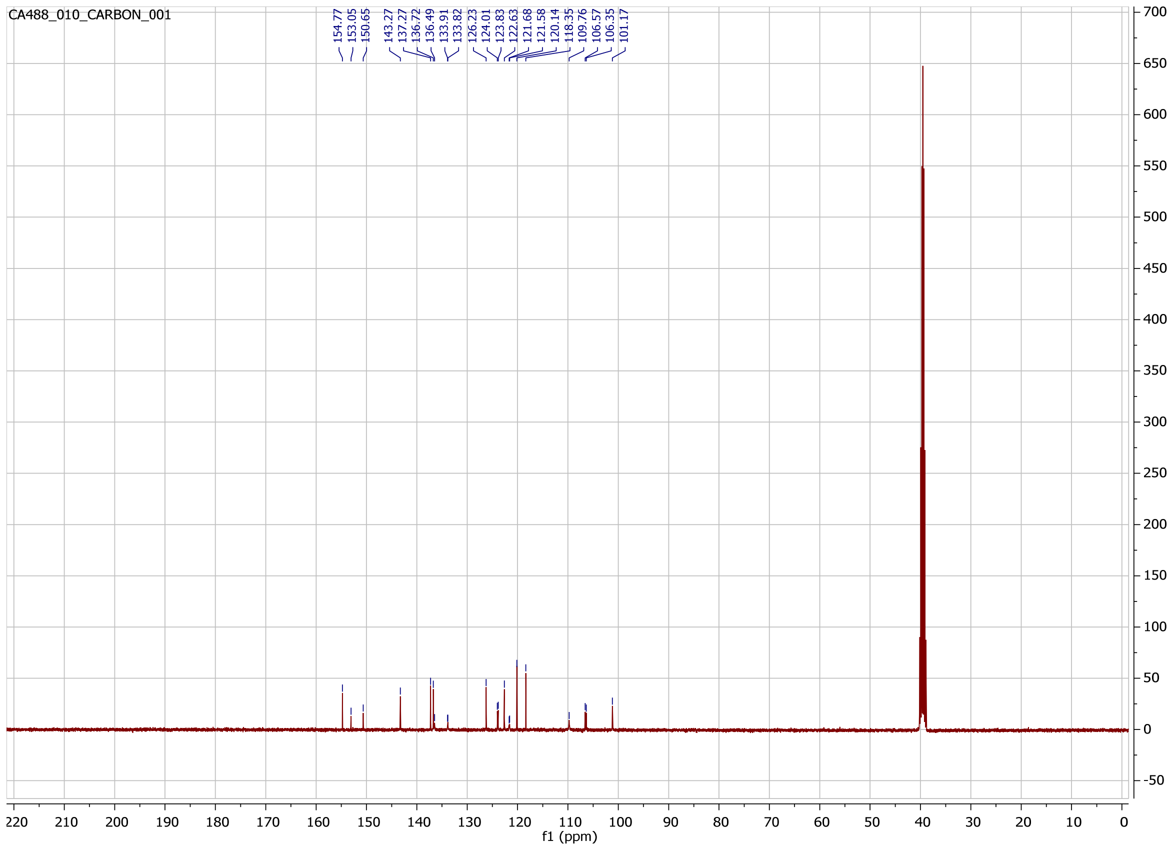
CA488_010_CARBON_001
154.77
153.05
150.65
143.27
137.27
136.72
136.49
133.91
133.82
126.23
124.01
123.83
122.63
121.68
121.58
120.14
118.35
109.76
106.57
106.35
101.17
f1 (ppm)

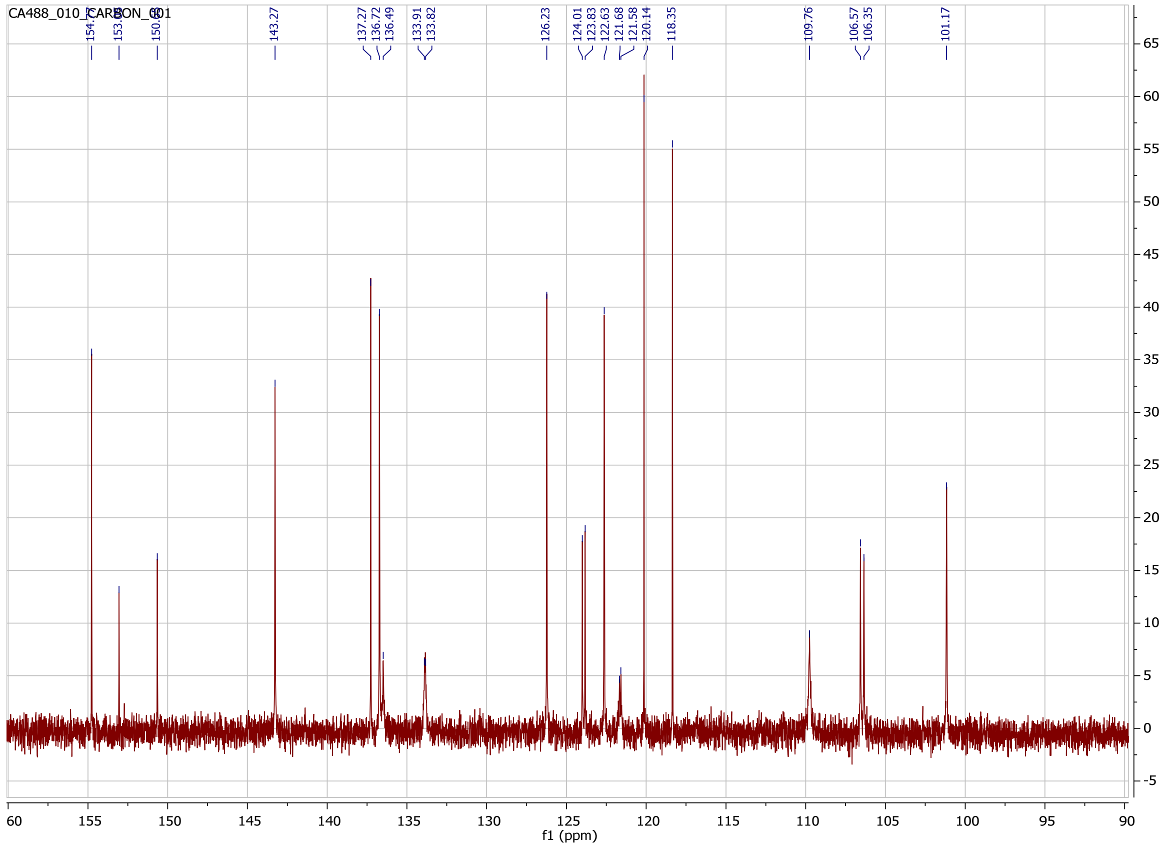
CA488_010_CARBON_001
143.27
137.27
136.72
136.49
133.91
133.82
126.23
124.01
123.83
122.63
121.68
121.58
120.14
118.35
109.76
106.57
106.35
101.17
f1 (ppm)

6-[(6-bromoquinolin-4-yl)amino]-2,3-dihydro-1*H*-isoindol-1-one (**52**)

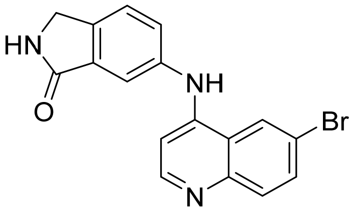

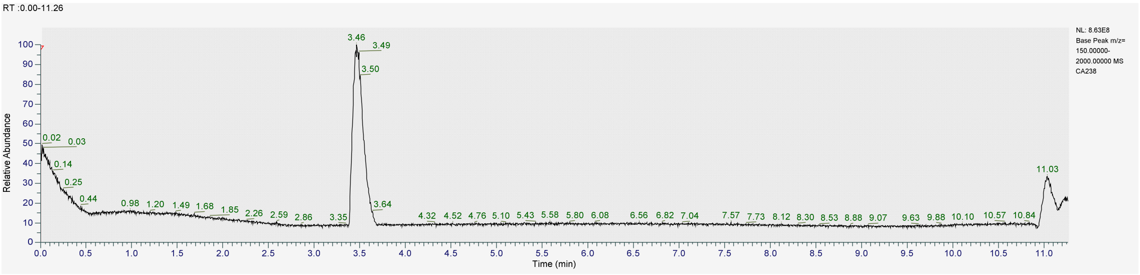

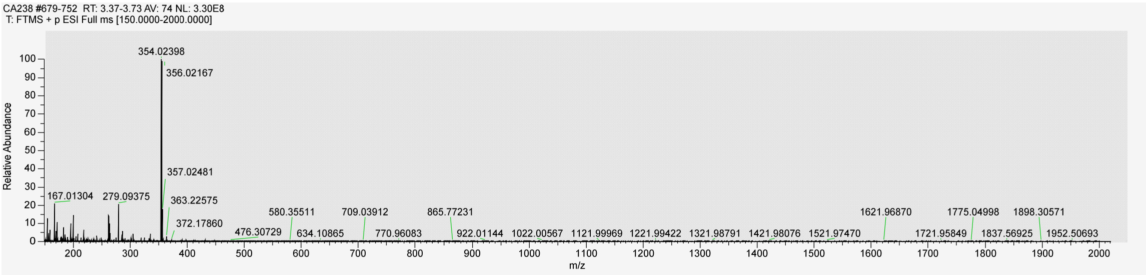

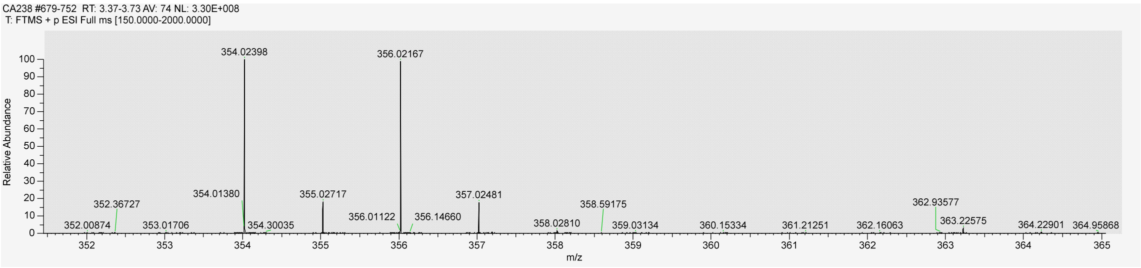

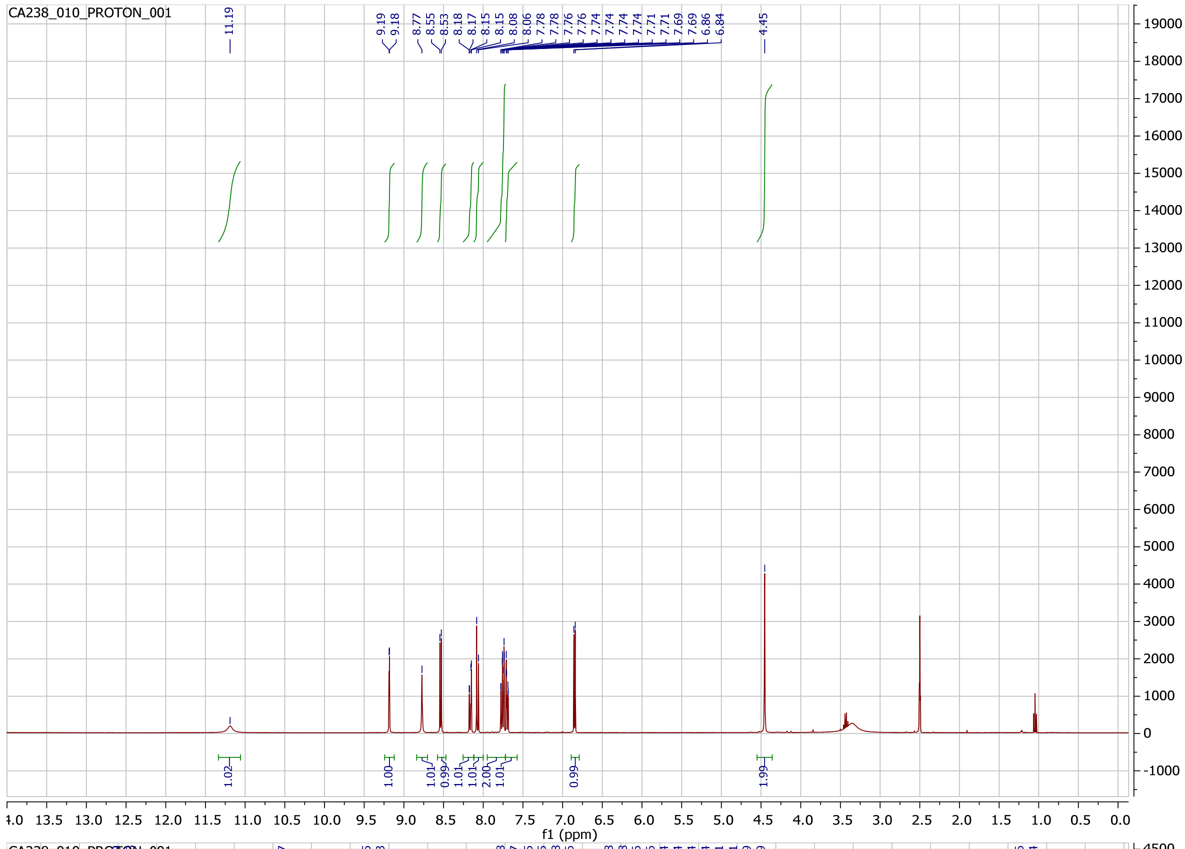
CA238_010_PROTON_001
f1 (ppm)

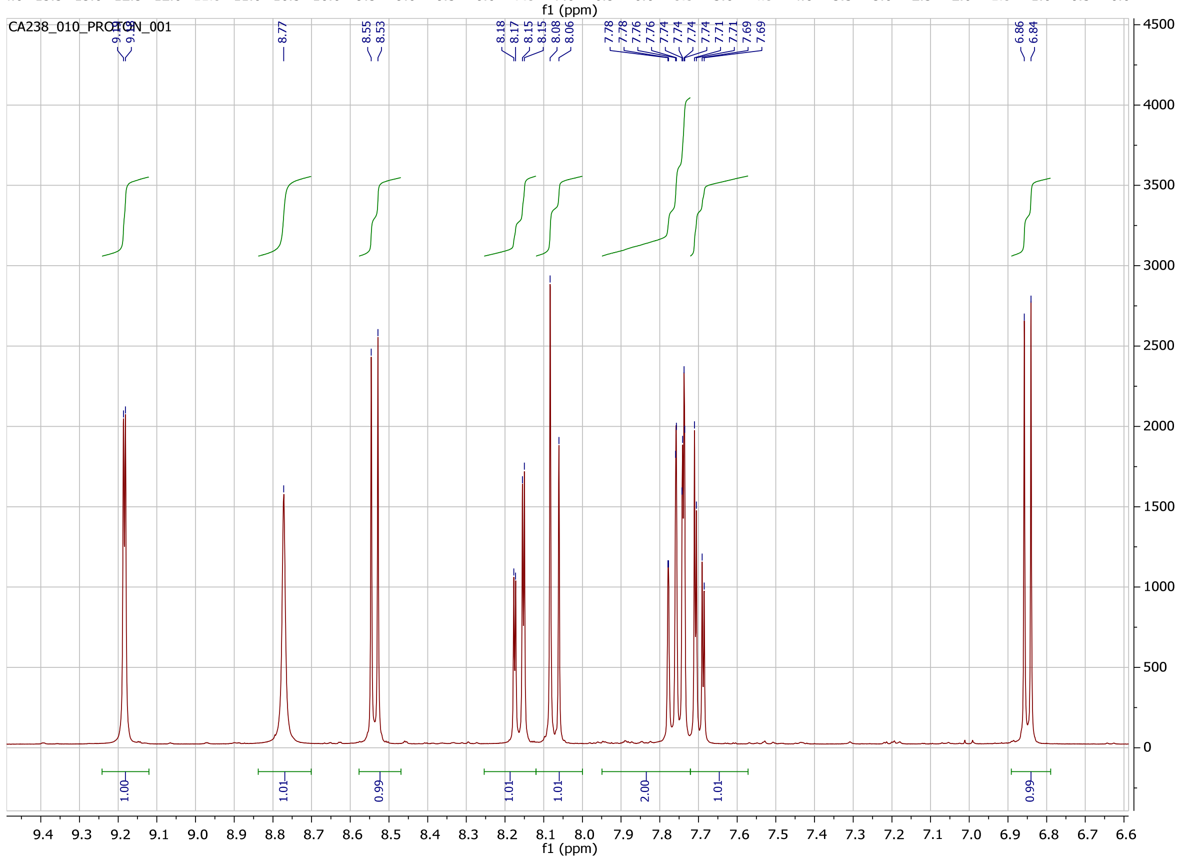
CA238_010_PROTON_001
f1 (ppm)

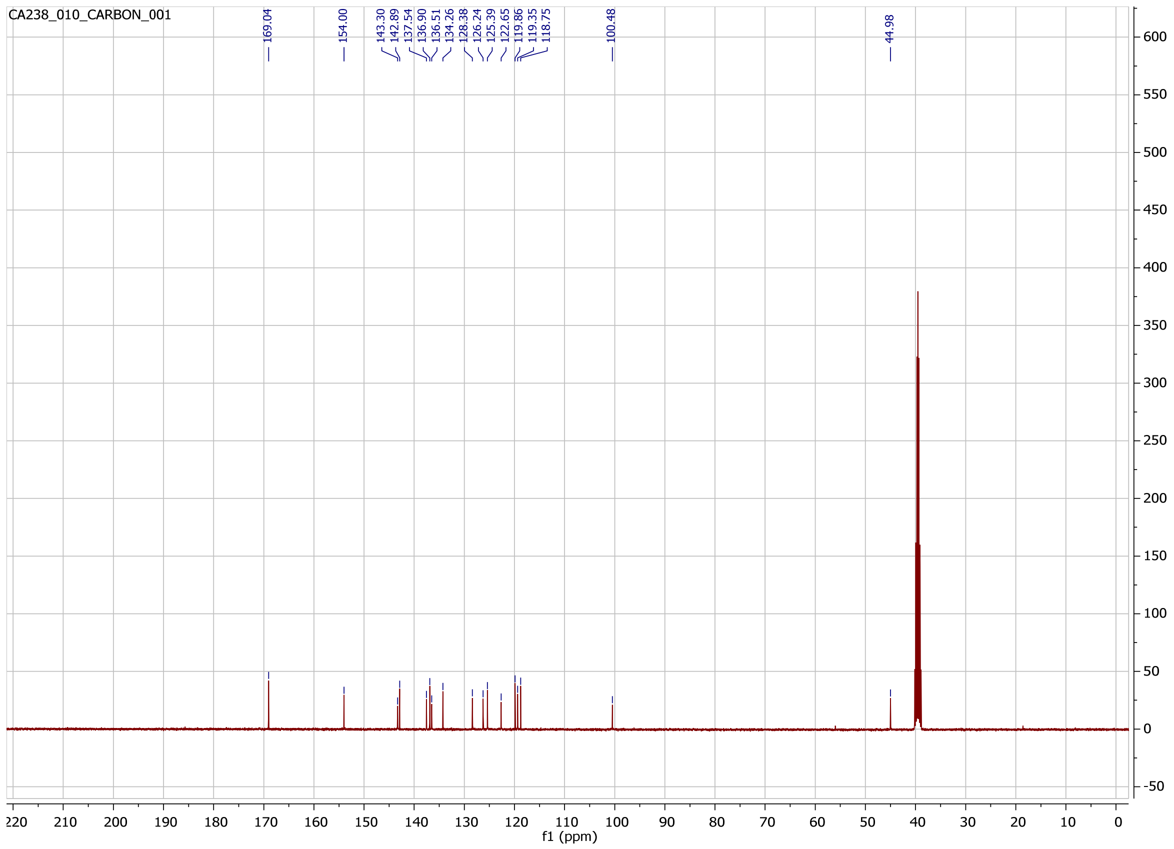
CA238_010_CARBON_001
169.04
154.00
143.30
142.89
137.54
136.90
136.51
134.26
128.38
126.24
125.39
122.65
119.86
119.35
118.75
100.48
44.98
f1 (ppm)
